# Catalyst Engineering for Photocatalytic Hydrogen Peroxide Production: State-of-the-Art Progress and Future Perspectives

**DOI:** 10.3390/nano16080466

**Published:** 2026-04-15

**Authors:** Yangyulu Huang, Shurui Cheng, Qixuan Chi, Wenjun Jiang

**Affiliations:** Department of Materials Science and Engineering, College of Transportation Engineering, Dalian Maritime University, Dalian 116026, China; phantomhive@dlmu.edu.cn (Y.H.); shuruicheng@dlmu.edu.cn (S.C.); chiqixuan77@dlmu.edu.cn (Q.C.)

**Keywords:** photocatalysis, hydrogen peroxide, catalyst design, charge separation, light absorption, sustainable oxidation

## Abstract

Hydrogen peroxide (H_2_O_2_) plays a vital role as an eco-friendly oxidizer, extensively used in environmental cleanup, energy transformation, and organic production. Nonetheless, the conventional method of creating anthraquinones is intricate, resulting in significant energy and ecological costs, which calls for the development of more eco-friendly and efficient substitute technologies. The article methodically examines the reaction processes and methods for improving efficiency in photocatalytic H_2_O_2_ generation in the past few years. This review summarizes the design principles and key structural features of various novel catalytic materials, focusing on light absorption, charge separation and migration, surface redox reactions, and enhanced mass transfer. Approaches such as expanding the range of bandgap absorption, building conjugated structures, and incorporating metal nanoclusters can significantly enhance the efficiency of light absorption. In the charge separation process, constructing built-in electric fields at the interfaces of heterojunctions, homojunctions, and Schottky junctions is crucial for improving reaction efficiency. Additionally, defect engineering may encourage targeted carrier movement and minimize recombination. The review highlights the latest advancements in enhancing selectivity and reducing H_2_O_2_ breakdown in surface redox reactions, achieved by regulating active sites, introducing new functional groups, and developing dual-channel reaction pathways. Furthermore, constructing three-phase interfaces, regulating asymmetric wettability, and designing cyclic/flow reactors provide innovative engineering solutions to address the challenges of insufficient oxygen supply and large-scale continuous production. Ultimately, the potential for producing H_2_O_2_ in photocatalytic systems is detailed.

## 1. Investigative Background

The main uses of hydrogen peroxide include its oxidation capabilities and eco-friendliness, and it is widely recognized as a good oxidizer. It is applied in the sanitizing and cleaning of wounds in the fields of health and hygiene. Hydrogen peroxide is applied in the industry for the bleaching of paper and textiles. In addition, in food engineering, it is applied for the bleaching of food products. Furthermore, in the field of environmental conservation, H_2_O_2_ has a major application in the treatment and disposal of wastewater. In the new generation of energy, H_2_O_2_ fuel cells allow electrochemical reactions to occur without the use of a catalyst. In the conventional proton exchange membrane fuel cell, H_2_O_2_ is an auxiliary agent. As an ideal cleaning oxidizing agent, H_2_O_2_ has a standard reduction potential as high as 1.8 V, which gives it extremely strong oxidizing power; moreover, its reaction product is merely water, so it does not cause secondary pollution. It is used as a green oxidizing agent in organic synthesis reactions and other applications [1].

The traditional method for synthesizing hydrogen peroxide involves the utilization of the anthraquinone process. During this transformation, 2-alkylanthraquinone reacts with hydrogen gas under the influence of a palladium-based catalyst, leading to the formation of 2-alkylanthrahydroquinone. The latter is then oxidized using oxygen to form hydrogen peroxide. The equation represents the overall reaction: H_2_ + O_2_ → H_2_O_2_ [2]. The autoxidation of anthraquinone is the most prominent technique to produce hydrogen peroxide, which offers many advantages, such as a high yield of H_2_O_2_ per cycle, relatively mild reaction conditions, and the possibility of continuous production. However, there remain significant inherent shortcomings. The total energy consumption for producing 1 tons of H_2_O_2_ is as high as 2.9–5.7 GJ, and the production of every 1 kg of H_2_O_2_ is accompanied by emissions of approximately 1.33 kg of carbon dioxide equivalent, resulting in annual CO_2_ emissions from this industry worldwide exceeding 2.8 Mt [3]. Empirical studies on life cycle assessment (LCA) have quantitatively demonstrated that the anthraquinone process accounts for over 95% of global hydrogen peroxide (H_2_O_2_) production. The hydrogenation step, due to its reliance on expensive platinum-group metal catalysts and its high thermal energy requirements, accounts for 22.2% of the process’s total Global Warming Potential (GWP); whereas catalyst loss, cooling water circulation and waste liquid treatment collectively contribute approximately 3.9% of the environmental impact, whilst also facing issues such as the need for solvents and hydrogenation catalysts, the occurrence of side reactions, the need to separate organic impurities to purify H_2_O_2_, and catalyst loss [4,5].

Conversely, the photocatalytic production of hydrogen peroxide employs semiconductor materials to absorb light energy, facilitating the catalytic reaction between oxygen and water to generate H_2_O_2_. This process typically involves three distinct stages: the absorption of light and generation of charge carriers, the separation and transfer of these charges, and finally, the surface redox reactions. The photocatalytic process exhibits distinct characteristics when compared to the anthraquinone auto-oxidation process, primarily due to its utilization of H_2_O and O_2_ as reactants, elimination of H_2_ or organic solvents, and reliance on solar energy as the driving force. The photocatalytic process represents a low-pollution methodology that avoids the release of any toxic or hazardous substances. The sole product generated, H_2_O_2_, aligns with the principles of green and sustainable environmental practices. This procedure can be performed at ambient temperature, thereby simplifying the operational complexity.

**Scheme 1:**
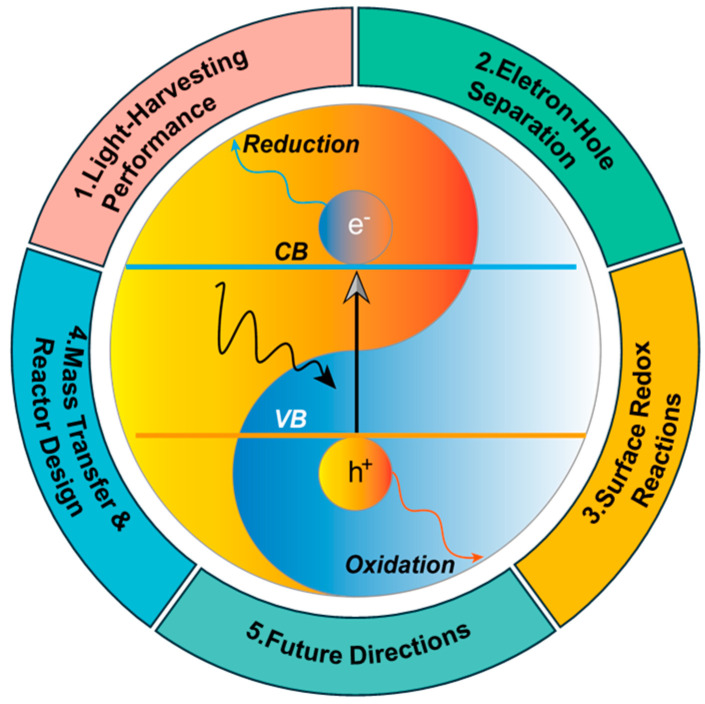
Catalyst engineering for photocatalytic hydrogen peroxide production.

Nevertheless, the photocatalytic production of hydrogen peroxide still encounters numerous challenges and limitations. For example, most photocatalysts have a narrow range of absorption in the solar spectrum and therefore cannot effectively use sunlight for catalysis; charge carriers, such as electrons and holes, are difficult to separate due to their strong tendency to recombine; and there are problems such as instability in the photocatalysts and limited active sites. This paper is a summary of some recent literature on solutions to the problems described above. Scheme 1 shows that there are four types of solutions: those for improving light harvesting, for improving electron–hole pair separation, for improving redox reactions, and for reactor design. Suggestions for the promotion of photocatalysis are also included [6].

## 2. Mechanisms for Enhancing Photocatalytic Performance

Photocatalysts are well known to be crucial for the production of hydrogen peroxide, and new materials have shown promising results in the fabrication of photocatalysts [7]. The classification of new photocatalysts for the production of hydrogen peroxide is described in the following subsection.

### 2.1. Light Absorption and Charge Generation—Enhancing Light-Harvesting Performance

**Figure 1:**
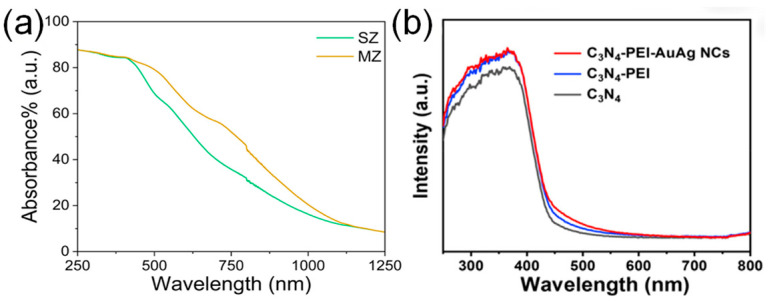
(**a**) Ultraviolet–visible diffuse reflectance spectra (DRS) of SZ and MZ [8]. Copyright 2025, Elsevier B.V. (**b**) Ultraviolet–visible DRS spectra [9]. Copyright 2024, KeAi.

The first step in photocatalysis is photoexcitation, which emphasizes the significance of efficient light harvesting. Li et al. successfully synthesized benzothiazole (SZ)- and benzimidazole (MZ)-based porous organic polymers (POPs) using a one-step method at room temperature. By adopting a conjugated framework strategy and atomic-level electronic engineering, they were able to successfully broaden the light absorption range of POPs to cover the whole visible region. This breakthrough allowed SZ and MZ to reach H_2_O_2_ photocatalytic production rates of 3109 μmol g^−1^h^−1^ and 2700 μmol g^−1^h^−1^ in pure water, respectively, outperforming most of the currently reported POPs-based catalysts. From the analysis of the UV–Vis diffuse reflectance spectroscopy (DRS) spectra (Figure 1a), SZ has a light absorption range of 400–650 nm, while MZ has an absorption range of 400–620 nm. Both SZ and MZ have broader absorption ranges, which are 50–100 nm wider than those of amorphous POPs. With a wavelength of 500 nm, the SZ and MZ samples have absorptivities of 0.87 and 0.92, respectively, relative to a BaSO_4_ reference, which is 1.9 to 2.0 times higher than the absorptivity of POPs without conjugated structures (0.45) [8]. Although SZ and MZ exhibit excellent photocatalytic efficiency, their near-infrared light capture efficiency remains negligible. Zhen and colleagues achieved the chemical integration of luminescent gold-silver nanocrystals (AuAg-NCs), stabilized by glutathione (GSH) ligands, with polyethyleneimine (PEI)-functionalized C_3_N_4_ via a crosslinking approach utilizing 1-(3-dimethylaminopropyl)-3-ethylcarbodiimide hydrochloride (EDC) and N-hydroxysuccinimide (NHS). This step brings highly luminescent AuAg-NCs, which can improve the ability of C_3_N_4_ to absorb visible light. Figure 1b illustrates the UV–Vis–DRS analysis, indicating that the absorption cutoff wavelength for pristine g-C_3_N_4_ is 500 nm, whereas for C_3_N_4_-PEI-AuAg-NCs, it extends to 650 nm. The intensity of absorption within the range of 400–800 nm is increased by 2.3-fold, making it possible for C_3_N_4_-PEI-AuAg-NCs to expand the range of light absorption [9] and make full use of near-infrared light. Compared with the original C_3_N_4_ (23 μM), the concentration of H_2_O_2_ produced in pure water is increased by 3.5-fold to 82 μM. Metal nanoclusters exploit the quantum confinement effect to transform metals from simple plasmonic absorbers into molecular-like absorbers capable of multiple discrete energy level transitions. This not only overcomes the bandgap limitations of semiconductors but also significantly enhances absorption intensity in the visible light region through the ligand-to-metal charge transfer (LMCT) effect and excellent interfacial charge transfer capabilities [10].

However, improvements in photostability are necessary. Another example is the inter-resorcinol-formaldehyde resin/carbon-supported Ta-N_2_O single-atom catalyst (RF/C-TaSA), which can capture a broad spectrum of light up to >932 nm [11]. By precisely controlling the ratio of two aldehyde monomers, toluene-2,5-dicarboxaldehyde (TA) and 2,5-bis(thien-2-yl)-toluene-2,5-dicarboxaldehyde (DTTA), and reacting them with 2,4,6-trimethyl-1,3,5-triazine (TMT), a high-performance TA/DTTA-2-TMT photocatalyst was developed. Under the optimal ratio conditions, the TA/DTTA-2-TMT photocatalyst exhibited a H_2_O_2_ generation rate of 3451 μmol g^−1^h^−1^ in pure water, ambient air, and under 100 mW cm^−2^ illumination. The integration of DTTA units substantially extended the material’s light absorption spectrum. This ratio-controlled preparation method presents a novel approach for synthesizing photocatalysts [12].

### 2.2. Electron–Hole Separation

During the catalytic formation of H_2_O_2_, it is imperative for electrons (e^−^) and holes (h^+^) to transfer to the catalyst’s surface, thereby participating in the catalytic reaction. However, in the process, there are high possibilities of recombination of the photo-generated electrons and holes either in the bulk or at the surface, which causes a reduction in the efficiency of charge separation in the conventional materials [13].

#### 2.2.1. Enhancing the Built-In Electric Field

**Figure 2:**
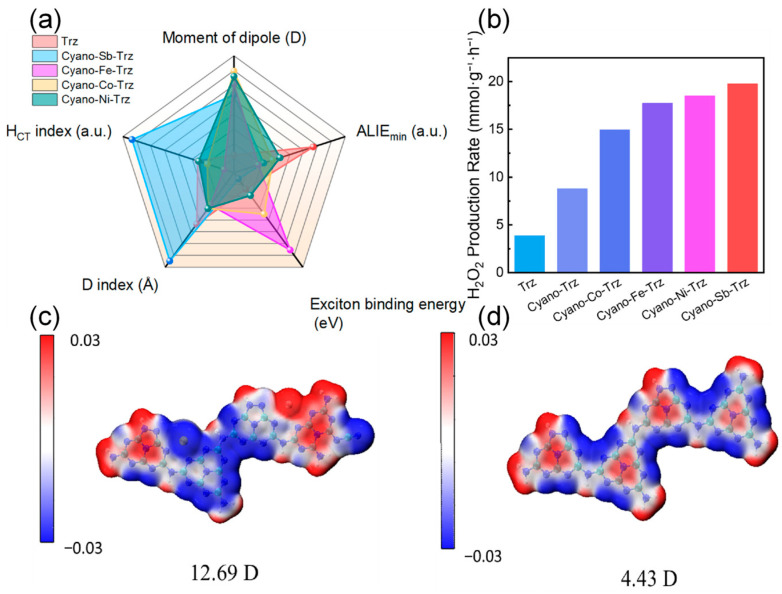
(**a**) Dipole moments, exciton binding energies, average local ionization energies, electron–hole centroid distances, and H indexes of charge transfer of different catalysts. (**b**) H_2_O_2_ production rate of Trz and Cyano-X-Trz in a 10% BA solution. (**c**) Electrostatic potential energy diagram of the basic structural unit of Cyano-Sb-Trz; (**d**) Electrostatic potential energy diagram of the basic structural unit of Trz [15]. Copyright 2025, Elsevier Ltd.

A critical issue in photocatalysis lies in the rapid recombination of photoexcited charge carriers, specifically electron–hole pairs. The inherent electric field serves as a crucial mechanism to address this challenge, efficiently directing the movement of charge carriers through a self-organized potential difference [14]. Multidimensional electronic structure parameters obtained from density functional theory (DFT) calculations reveal the mechanism by which co-doping with cyano and metal modifies the carrier behavior in carbon nitride. The modification strategy involving co-doping of the cyano group with Sb and K elements can effectively regulate key parameters of the material, including the molecular dipole moment, exciton binding energy, average local ionization energy, electron–hole centroid distance, and H indexes of charge transfer. As shown in Figure 2a, compared with the Trz, the molecular dipole moment of Cyano-Sb-Trz is significantly enhanced, the exciton binding energy is substantially reduced, whilst the electron–hole centroid distance increases significantly and H indexes of charge transfer rises markedly. This fundamentally lowers the dissociation energy barrier of photo-generated excitons and enhances the driving effect of the intramolecular electric field, providing an intrinsic structural basis for suppressing carrier recombination and improving carrier mobility. The results of the molecular electrostatic potential distribution calculations further clarify the structural essence of the directional migration and spatial separation of photo-generated carriers. As shown in Figure 2c, the Cyano-Sb-Trz material, modified by co-doping with Sb and K and cyano groups, exhibits significant spatial charge polarization: The cyano groups and Sb elements act as strong electron-withdrawing sites, causing electrons to accumulate in their vicinity, whilst holes concentrate near the K sites. This successfully constructs spatially separated redox active sites, providing sufficient driving force for the directional and rapid migration of photo-generated carriers, thereby effectively suppressing carrier recombination.

In stark contrast, as shown in Figure 2d, the unmodified pristine Trz material exhibits a uniform molecular charge distribution with no distinct regions of positive or negative potential polarization, and is thus unable to generate an effective driving force for carrier migration. The electron–hole pairs generated by photoexcitation readily recombine within the bulk phase, making it difficult to form effective carriers capable of participating in surface catalytic reactions; this is the primary reason for its extremely low intrinsic photocatalytic activity. Under simulated visible light irradiation (λ > 420 nm), catalysts modified with various metal dopants all demonstrated an improvement in H_2_O_2_ production performance compared to the Trz-based material. As shown in Figure 2b, the H_2_O_2_ yield of the pristine Trz is only 3.8 mmol g^−1^h^−1^, whereas the yield of the cyano-functionalized Cyano-Trz increases to 8.8 mmol g^−1^h^−1^, demonstrating the key promotional effect of cyano group introduction on photocatalytic performance. Among these, the Sb-doped Cyano-Sb-Trz exhibited the optimal catalytic performance, with an H_2_O_2_ yield reaching 19.8 mmol g^−1^h^−1^, which is 5.2 times and 2.6 times that of Trz and Cyano-Trz, respectively, and outperformed other metal-doped reference samples such as Co, Fe and Ni, demonstrating that Sb doping has the most significant effect on enhancing the photocatalytic H_2_O_2_ production performance of Trz-based materials. To further achieve the aforementioned highly efficient charge separation, the researchers developed various macrostructural strategies, such as the creation of built-in electric fields and defect engineering, in addition to micro-scale intrinsic regulation [15].

The external power source is not involved in the creation of this inherent electric field. It is created by space charge layers due to p-n junctions, Schottky barriers, heterojunctions, or defects. It improves the photocatalytic efficiency in the following ways: it overcomes the Coulomb forces between carriers through directed electrostatic forces, efficiently promoting their separation to reduce recombination; it promotes the carrier transport from the interior of the catalyst to the surface based on the electric potential gradient, thus lowering the reaction resistance; it can also steer the carriers to desired reactions by designing the electric potential direction and magnitude to improve selectivity; it decreases the reaction’s activation energy through the polarization of reactant molecules and the enhancement of interfacial charge density [16]. In conclusion, the internal electric field plays an effective role in overcoming the bottlenecks of photocatalysis in the entire process, from carrier separation to transport and reaction. The internal electric field is a key route for improving the efficiency of water splitting reactions for hydrogen evolution and CO_2_ reduction.

**Figure 3:**
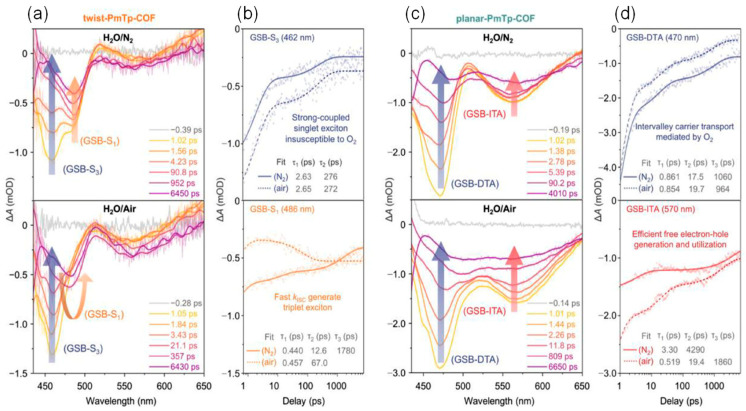
Configuration-dependent triplet exciton production (**a**) The fs-TAS spectra at different decay times, and (**b**) corresponding dynamics curves of GSB-S_3_ and GSB-S_1_ for twist-PmTp-COF in water with N_2_ and air atmosphere; (**c**) The fs-TAS spectra at different decay times, and (**d**) corresponding dynamics curves of GSB-DTA and GSB-ITA for planar-PmTp-COF in water with N_2_ and air atmosphere [18]. Copyright 2026, John Wiley & Sons, Ltd.

When studying built-in electric fields, the quantitative calculation and intuitive characterization of their intensity are key to elucidating charge transport mechanisms. Currently, researchers typically combine experimental observations with theoretical calculations to assess the intensity of built-in electric fields. The built-in electric field can be calculated using the formula $E\propto k\sqrt{\delta *V}$, which indicates that the strength of the built-in electric field is proportional to the surface charge density multiplied by the square root of the surface potential. The surface potential can be measured using a Kelvin probe force microscope [17]. Furthermore, transient absorption spectroscopy (TAS) and time-resolved photoluminescence spectroscopy (TRPL) enable the intuitive and quantitative tracking of the relaxation, transfer processes and lifetimes of excited-state electrons and holes. In recent years, researchers have extensively utilized these dynamic characterization techniques to demonstrate the role of structural design in promoting charge dynamics. For example, by comparing two covalent organic frameworks (COFs) with different topological structures, the differences in exciton evolution pathways can be clearly revealed. In the fs-TAS spectrum (Figure 3c) and kinetic decay curve (Figure 3d) of the planar configuration (planar-PmTp-COF), the photogenerated carriers exhibit extremely rapid recombination, with an average lifetime t of merely 0.861 ps. In contrast, the twist-PmTp-COF, constructed via topological regulation, exhibits distinct signal evolution in the spectrum (Figure 3a), and its kinetic curve (Figure 3b) confirms an extremely rapid intersystem crossing (ISC) process. This process enables the material to efficiently generate long-lived triplet excitons with lifetimes as high as 3830 ps, representing an improvement of several thousand times over planar structures. This extension of lifetimes on the picosecond to nanosecond scale provides an ample supply of long-lived active charges for the photolysis of H_2_O_2_, offering a kinetic explanation for the significant enhancement in catalytic performance [18].

Hence, creating and enhancing an intrinsic electric field offers a promising approach for improving the catalytic efficiency of our catalyst. We have reviewed some of the latest methods for enhancing built-in electric fields and have categorized them into the following three types.

(1) Constructing a heterojunction:

A heterojunction is a semiconductor material structure created through the interface bonding of two or more semiconductor materials that have different compositions, crystal structures, or phases. The basic principle of a heterojunction is based on taking advantage of the differences in the electronic structures of the materials on both sides of the interface to create the force for charge transfer [19].

Its primary role is to promote charge separation upon illumination. Upon photoexcitation of a single semiconductor material, valence band electrons transition to the conduction band, thereby generating electron–hole pairs simultaneously. The creation of a heterojunction leads to the formation of an energy level gradient and an internal electric field, which disturbs the equilibrium of randomly distributed charges. This facilitates the directional separation of electrons (e^−^) and holes (h^+^). Furthermore, the formation of a heterojunction structure significantly improves the stability of photocatalytic materials, thereby effectively addressing the stability challenges often encountered in photocatalysts primarily designed for enhanced light absorption efficiency [20].

**Figure 4:**
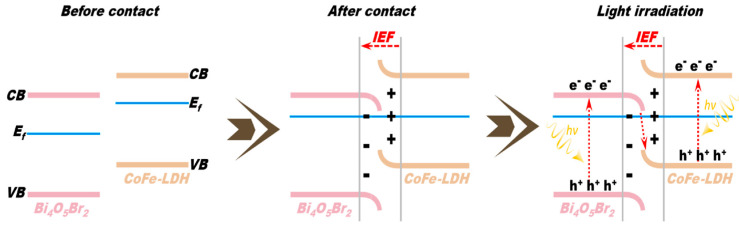
Schematic representation of the charge transfer mechanism between Bi_4_O_5_Br_2_ and CoFe-LDH [21]. Copyright 2019, John Wiley & Sons, Ltd.

As a typical type of heterojunction, the S-type heterojunction further optimizes this core mechanism: the built-in electric field not only achieves directional separation of charge carriers but also promotes the selective recombination of low-energy charge carriers in both semiconductors, retaining only high-energy electrons and holes with strong redox capabilities, thereby balancing separation efficiency and catalytic activity. Figure 4 illustrates this mechanism using CoFe-LDH/Bi_4_O_5_Br_2_ as an example: prior to contact, the Fermi levels and band structures of CoFe-LDH (work function 1.36 eV) and Bi_4_O_5_Br_2_ (work function 5.51 eV) are misaligned. Following contact, electrons migrate from CoFe-LDH to Bi_4_O_5_Br_2_, creating an intrinsic electric field directed from CoFe-LDH towards Bi_4_O_5_Br_2_; Under illumination, this electric field drives the recombination of low-energy electrons in the conduction band of Bi_4_O_5_Br_2_ with low-energy holes in the valence band of CoFe-LDH, whilst allowing high-energy electrons in the conduction band of CoFe-LDH and high-energy holes in the valence band of Bi_4_O_5_Br_2_ to participate in the reaction, thereby clearly revealing the core operational logic of the S-type heterojunction [21].

**Figure 5:**
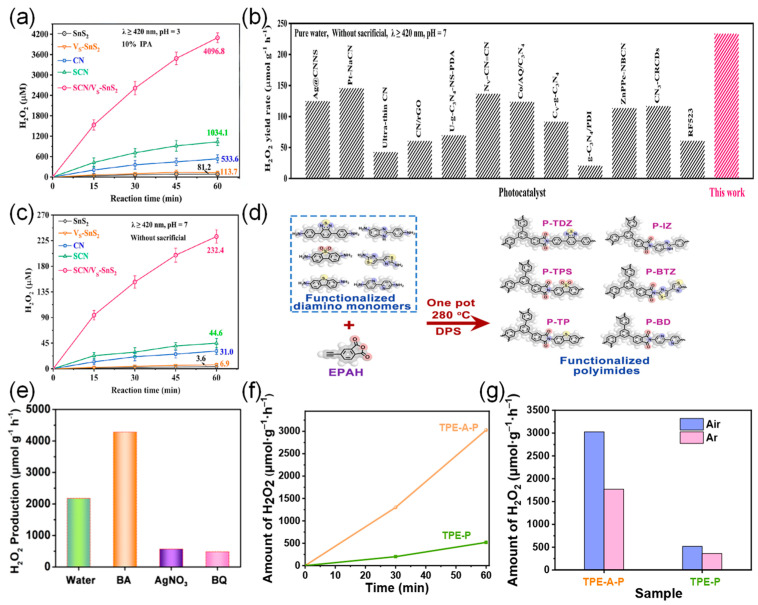
Yield evolution curves of prepared samples over time: (**a**) 10% IPA solution (pH = 3), (**b**) pure water system, comparing SCN/Vs-SnS_2_ with previously reported photocatalysts’ generation performance, (**c**) pure water (pH = 7) [34]. Copyright 2025, Elsevier B.V. (**d**) MPIs prepared via domino polymerization. (**e**) H_2_O_2_ generation of P-TDZ under different scavengers [35]. Copyright 2025, American Chemical Society. (**f**) Photocatalytic generation rates of TPE-A-P and TPE-P in open air and pure water without any additives. (**g**) Photocatalytic production of TPE-A-P and TPE-P in pure water under different atmospheric conditions [36]. Copyright 2024, MDPI AG.

Lang et al. successfully fabricated 2D/2D SCN/V_S_-SnS_2_ heterojunctions via a one-step chemical vapor deposition process, taking advantage of the similar growth temperatures of sulfur-doped carbon nitride and sulfur vacancies (V_S_-SnS_2_), which are between 350 and 500 °C. As illustrated in Figure 5a, the sacrificial agent isopropanol (IPA) facilitated the generation of approximately 4096.8 μmol of H_2_O_2_. The data presented in Figure 5b demonstrate that the SCN/V_S_-SnS_2_ heterojunction exhibits remarkable photocatalytic performance when contrasted with the majority of previously documented photocatalytic materials [22,23,24,25,26,27,28,29,30,31,32,33]. Nevertheless, in the scenario of pure water devoid of sacrificial agents, as illustrated in Figure 5c, the production of H_2_O_2_ is confined to a mere 232.4 μmol. This limitation poses significant challenges for eliminating sacrificial agents in practical applications [34]. Thus, minimizing the use of sacrificial agents via heterojunction engineering reduces the need for conventional, highly toxic sacrificial agents, such as methanol and isopropanol, and instead uses low-toxicity biodegradable co-reactants when necessary. A strategy that seeks to harmonize catalytic performance with environmental needs is a promising area of research. Xiong et al. prepared microporous polyimides (MPIs) with donor-acceptor (D-A) architecture through domino polymerization. Enhancements in the solvothermal method enable the preparation of a set of MPIs with diverse functional groups in only 0.5 h. The schematic diagram of MPIs with various functional units synthesized by domino polymerization is shown in Figure 5d. Of these, the thiazole-containing MPI (P-TDZ) has very strong visible light absorption and separation abilities due to its D-A structure. The curve presented in Figure 5e illustrates that, with the facilitation of phenylacetone, the H_2_O_2_ generation rate of P-TDZ attains 4280 μmol g^−1^h^−1^. It is noteworthy that, even without the presence of a sacrificial agent, a H_2_O_2_ production rate of 2177 μmol g^−1^h^−1^ can still be attained [35]. The structure of this catalyst reduces the dependence on sacrificial agents, thus improving its environmental sustainability. In a similar study, Liu et al. synthesized an organic polymer named terpolymer TPE-A-P, which has a donor–bridge–acceptor (D-B-A) structure similar to that of the donor-acceptor (D-A) structure. Through the Sonogashira-Hagihara coupling reaction involving tetraphenyl ethylene (TPE) as the electron donor, acetylene (A) serving as the linker, and pyrene (P) acting as the electron acceptor, the D-B-A molecular strategy with redox activity significantly reduces the exciton binding energy of the photocatalyst. This reduction effectively suppresses the recombination process between photogenerated holes and electrons. As shown in Figure 5f, the photocatalytic H_2_O_2_ production rate of TPE-A-P is 3028 μmol g^−1^h^−1^. As illustrated in Figure 5g, remarkably, even under anaerobic conditions, TPE-A-P still exhibits over 50% H_2_O_2_-generating activity [36].

**Figure 6:**
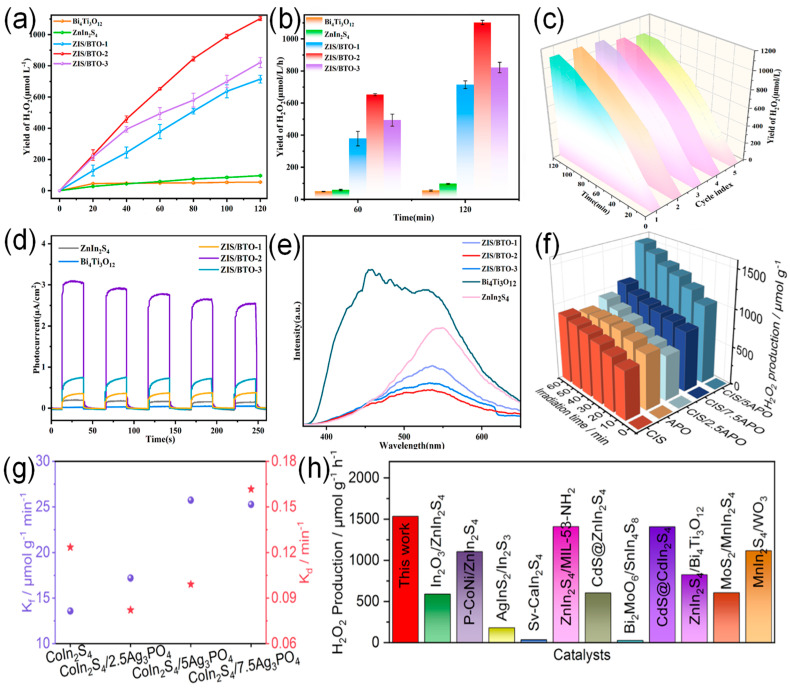
(**a**,**b**) Photocatalytic production under visible light irradiation (three experiments). (**c**) Cyclic photocatalytic production experiment of ZIS/BTO-2 composite. (**d**) Transient photocurrent measurement. (**e**) Photoluminescence spectrum [37]. Copyright 2025, Elsevier B.V. (**f**) Production rates of CoIn_2_S_4_ and CoIn_2_S_4_/Ag_3_PO_4_ under visible light irradiation. (**g**) Fitted production rate (K_f_) and decomposition rate (K_d_) for visible light production using catalysts with different composite ratios. (**h**) Comparative photocatalytic production performance of various sulfide-based photocatalysts [38]. Copyright 2025, Elsevier B.V.

Hong et al. synthesized a Zn In_2_S_4_/Bi_4_Ti_3_O_12_ (ZIS/BTO) composite photocatalyst by growing ZnIn_2_S_4_ on Bi_4_Ti_3_O_12_ nanosheets. This catalyst efficiently generates H_2_O_2_ from water and oxygen through the oxygen reduction reaction (ORR) and oxygen evolution reaction (OER) pathways. As illustrated in Figure 6a,b, the H_2_O_2_ yield reached 1102 μmol L^−1^ in a 10% ethanol solution. Notably, ethanol did not act as a conventional sacrificial agent, and ZIS/BTO demonstrated exceptional stability, maintaining a yield exceeding 1000 μmol L^−1^ after five operational cycles (Figure 6c). The heterojunction of BiOCl/COF(TFPB-BPY)-S was successfully fabricated via a facile in situ growth approach. When the mass fraction of BiOCl in the composite was modified to 20%, referred to as the 20BTB composite, its peak photocatalytic output reached 4088.46 μmol g^−1^h^−1^, as illustrated in Figure 6d,e. This value represents an enhancement of 2.8-fold compared to COF and a significant increase of 19.2-fold relative to pure BiOCl [37]. Each of these structures is based on an S-type charge transfer process. By controlling the band gap and work function differences between the two components, they build an interleaved band structure, which is described as an “oxidation-type semiconductor-reduction-type semiconductor.” The S-type heterojunction structure is used to overcome the shortcomings of the individual materials. The “in situ growth” approach is used to build a tight interface. This approach establishes charge transfer pathways, thereby promoting the separation of photo-generated carriers. Nevertheless, with the increase in BiOCl content within the composite, the generation of H_2_O_2_ experiences an initial rise followed by a subsequent decrease. This reduction could be ascribed to enhanced light attenuation, obstruction of active sites, and restrained charge carrier transfer due to material aggregation, which ultimately impairs photocatalytic performance. Similarly, Zhen et al. employed CoIn_2_S_4_ (E_CB_ ~ −0.893 eV, E_VB_ ~ 0.523 eV) and Ag_3_PO_4_ (E_CB_ ~ −0.227 eV, E_VB_ ~ 2.453 eV) with band structures favorable for H_2_O_2_ production to construct a heterojunction for the CoIn_2_S_4_/Ag_3_PO_4_-S system. Through band structure modification, they optimized the heterojunction and obtained a H_2_O_2_ yield of 1534.78 μmol g^−1^. As shown in Figure 6f,g, without a sacrificial agent, the H_2_O_2_ yield of CoIn_2_S_4_ was 828.84 μmol g^−1^. Nevertheless, an overabundance of Ag_3_PO_4_ similarly resulted in a decline in H_2_O_2_ production (Figure 6h) [38].

**Figure 7:**
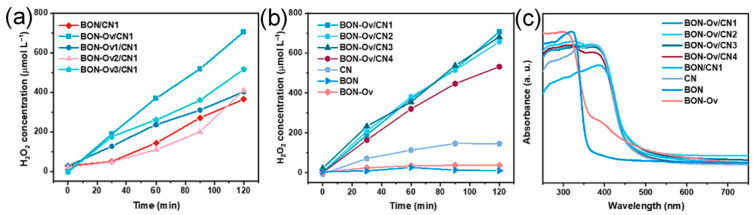
(**a**,**b**) Photocatalytic H_2_O_2_ production curves of the prepared samples; (**c**) UV–visible diffuse reflectance spectra [39]. Copyright 2024, Elsevier B.V.

Another method that can be employed to improve charge transfer at the heterojunction interface is based on the regulation of the vacancy-induced internal electric field (IEF). The methodology employs a composite material comprising g-C_3_N_4_ and Bi_2_O_3_(NO)_3_(OH), which is modified with oxygen vacancies (Ovs), referred to as BON-Ov/CN. As illustrated in Figure 7a,b, the composite material exhibits a photocatalytic H_2_O_2_ production efficiency of 706.2 μmol L^−1^. Moreover, an efficient approach to improve the utilization of visible light enables BON-Ov to show strong tailing absorption. As shown in Figure 7c, the light absorption range of BON-Ov is extended to 520 nm [39].

In the most recent studies on heterojunctions, a titanium-based metal-organic framework (MOF) and sodium-doped carbon nitride has been synthesized as an S-type heterojunction photocatalyst. The composite material shows improved charge separation efficiency by incorporating Na-CN with strong redox capabilities [40]. The modification of MgIn_2_S_4_ nanosheets onto hollow spherical carbon-based organic frameworks (COFs) to construct MgIn_2_S_4_/COF (MC) S-type heterojunctions facilitates the simultaneous occurrence of continuous 2e^−^ O_2_ reduction and 4e^−^ H_2_O oxidation [41]. In addition, the COF/COF II heterojunction TpPa/TpDz shows a near-complete spatial separation of the highest occupied molecular orbitals (HOMO) and lowest unoccupied molecular orbitals (LUMO), facilitating the directional transport of the photoexcited carriers [42]. Furthermore, the Z-scheme heterojunction, formed through the integration of I^−^/K^+^ doped g-C_3_N_4_ with the metal-organic framework NH_2_-UiO-66, exhibits superior catalytic performance for O_2_ reduction to H_2_O_2_ while significantly enhancing the selectivity of the 2e^−^ ORR [43,44].

**Figure 8:**
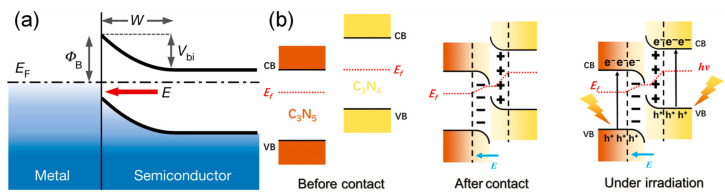
(**a**) Schematic of a Schottky junction showing the potential variation in the depletion region, where *E_F_* is the Fermi level, *Φ_B_* is the barrier height, *V_bi_* is the built-in potential, *W* is the depletion region and *E* denotes the electric field [47]. Copyright 2020, Springer Nature Limited. (**b**) Formation of the CNHJ S-scheme heterojunction and the proposed charge transfer and separation mechanism [49]. Copyright 2025, John Wiley & Sons, Ltd.

(2) Forming a Schottky junction:

Schottky junction represents a structure of the metal–semiconductor interface with close contact between the metal and the semiconductor, having unidirectional conductivity (rectifying) properties. The essence of the junction is the Schottky barrier. The Schottky barrier represents an interfacial potential barrier that governs the flow of charge carriers, thereby differentiating it from Ohmic contacts, which are non-rectifying metal–semiconductor junctions [45]. From a basic point of view, the main difference between metals and semiconductors is based on their Fermi levels. Metals have a relatively high concentration of free electrons, and their Fermi level is usually below that of n-type semiconductors, where electrons are the majority carriers and the Fermi level is close to the conduction band [46]. When two materials are brought close together without much impurity or defect at the interface, electrons tend to move spontaneously from the conduction band of the n-type semiconductor, with a higher Fermi level, to the metal, which has a lower Fermi level. As a result of the movement of electrons, the semiconductor loses electrons, leading to a positive charge on the donor ions and the creation of a weak ‘space charge region’ or depletion region. On the other hand, the metal gains electrons and becomes negatively charged. The interface between the semiconductor and the metal exhibits an intrinsic electric field, which arises from the spatial separation of positive and negative charge regions and is directed toward the metallic side. The subsequent electron flow is impeded by this electric field. Finally, diffusion and repulsion forces reach a state of equilibrium, creating a stable energy barrier at the junction, which is called the Schottky barrier. The magnitude of the Schottky barrier is primarily determined by the metal’s work function, which represents the energy required to extract an electron from the metal, as well as the semiconductor material’s electron affinity. For n-type semiconductors, the barrier height is approximately equal to the metal’s work function minus the semiconductor’s electron affinity. Figure 8a visually illustrates the spatial distribution of the aforementioned core elements (depletion region, Fermi level, barrier, built-in electric field and potential), clearly revealing the core mechanism of the Schottky junction from contact to equilibrium [47].

**Figure 9:**
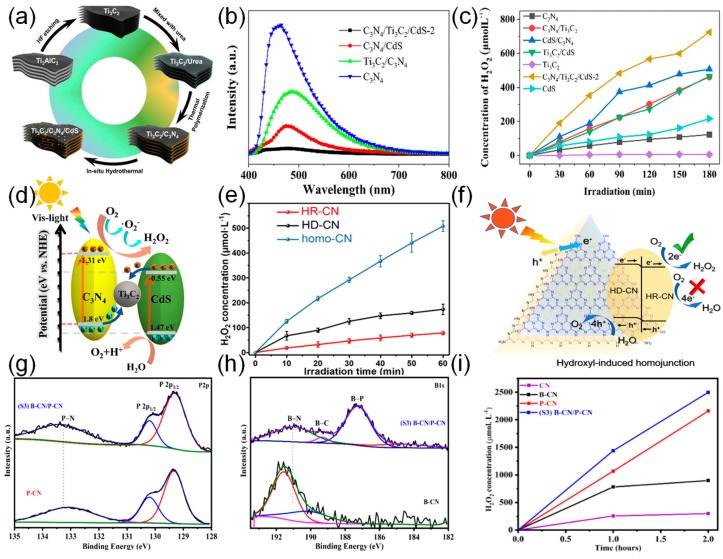
(**a**) Preparation process of C_3_N_4_/Ti_3_C_2_/CdS. (**b**) Photoluminescence (PL) spectra of C_3_N_4_, Ti_3_C_2_/C_3_N_4_, C_3_N_4_/CdS, and C_3_N_4_/Ti_3_C_2_/CdS-2. (**c**) Photocatalytic H_2_O_2_ generation rates of C_3_N_4_, Ti_3_C_2_, CdS; C_3_N_4_/Ti_3_C_2_, C_3_N_4_/CdS; Ti_3_C_2_/CdS; and C_3_N_4_/Ti_3_C_2_/CdS-2. (**d**) Photocatalytic mechanism of C_3_N_4_/Ti_3_C_2_/CdS [48]. Copyright 2023, The Royal Society of Chemistry. (**e**) The H_2_O_2_ yield of HR-CN, HD-CN and homo-CN (1 mg/mL) with 10% ethanol (**f**) Mechanism for the improved photocatalytic H_2_O_2_ generation over the hydroxyl-induced homojunction [52]. Copyright 2023, Elsevier B.V. (**g**,**h**) High-resolution XPS spectrum of P 2p and Bi 1s. (**i**) Photocatalytic performance under LED_365nm_ of all samples [51]. Copyright 2025, American Chemical Society.

C_3_N_4_/Ti_3_C_2_/CdS was prepared using the process shown in Figure 9a. When the mass ratio of CdS to C_3_N_4_/Ti_3_C_2_ was 1 wt%, 2 wt%, they were designated as C_3_N_4_/Ti_3_C_2_/CdS-1, C_3_N_4_/Ti_3_C_2_/CdS-2, and C_3_N_4_/Ti_3_C_2_/CdS-3, respectively. In this ternary material, the addition of Ti_3_C_2_, C_3_N_4_, and CdS enables the formation of a double Schottky junction structure, which enhances the H_2_O_2_ production rate. Under visible light irradiation, the H_2_O_2_ production rate reaches as high as 256 mmol L^−1^h^−1^, as shown in Figure 9b. Among these, C_3_N_4_/Ti_3_C_2_/CdS-2 exhibits the lowest fluorescence peak intensity, while Figure 9c indicates that it has the highest photoelectron transfer efficiency. Throughout the photocatalytic system, the transfer of photogenerated carriers follows a Z-shaped transfer path, which facilitates the effective spatial separation of photogenerated electrons and holes. Figure 9d illustrates the photocatalytic mechanism of C_3_N_4_/Ti_3_C_2_/CdS [48].

(3) Forming a Homogeneous Junction:

Homojunction: A P-type junction created by doping the same semiconductor material with different dopants to form P-type and N-type regions is known as a homojunction. The main principle of a homojunction is based on the interaction between the diffusion of carriers and electric fields. At equilibrium, the variation in charge carrier concentration between the P-type and N-type regions induces electron diffusion from the N-type region towards the P-type region. On the other hand, holes diffuse from the P-type region to the N-type region. Following recombination, stationary positive and negative ions remain on either side of the interface, forming depletion zones that generate an intrinsic electric field directed from the N-type to the P-type region. This intrinsic electric field inhibits the diffusion of majority carriers while simultaneously facilitating the movement of minority carriers (electrons in P-type and holes in N-type) towards drift. At the point where diffusion and drift rates reach equilibrium, no net current flows across the junction, resulting in a stable equilibrium condition [50].

Upon the application of forward bias, the externally applied electric field counteracts the built-in electric field (BIEF), consequently diminishing both the intensity of the BIEF and the spatial extent of the charge region. This reduces the diffusion barriers for majority carriers. Electrons from the N-region and holes from the P-region can now diffuse in large numbers across the junction, acting as non-equilibrium carriers that recombine as they diffuse. The continuous supply of carriers in the semiconductor, maintained by an external power source, gives rise to a persistent forward current flow. With the augmentation of forward bias, the intensity of majority carrier diffusion correspondingly escalates, thereby leading to a rise in current flow.

Upon application of a reverse bias, the externally applied electric field aligns with the direction of the intrinsic BIEF, leading to an enhancement in both the magnitude of the BIEF and the spatial extent of the charge-depleted region. As a result, the junction region presents a significant barrier to the migration of majority carriers, causing the diffusion current to approach negligible levels. At this point, the drift component of the built-in electric field on minority carriers becomes more significant. The minority carriers can now cross the junction region, thus contributing to the reverse current. Nevertheless, due to the exceedingly low concentration of minority carriers, the reverse current diminishes and gradually reaches a stable state. When the reverse bias surpasses a specific critical threshold, the intrinsic electric field generates a significant quantity of new carriers, resulting in a sharp surge in reverse current and subsequently triggering reverse breakdown. If the current is not properly limited, it may lead to damage to the homojunction.

The S-type homojunction is a typical optimized variant of the homojunction, with its core mechanism further enhanced: The built-in electric field not only achieves the directional spatial separation of photo-generated carriers but also promotes the selective recombination of low-energy carriers in the two components of the homojunction, retaining only high-energy electrons and holes with strong redox capabilities. This enhances charge separation efficiency whilst avoiding defects commonly associated with heterojunctions, such as metal leaching, lattice mismatch and loss of redox potential. Figure 8b illustrates this mechanism using a C_3_N_5_/C_3_N_4_ polymer homojunction (CNHJ): The distribution of work functions and band offsets prior to contact; following contact, a directional built-in electric field is formed. Under illumination, this electric field drives the selective recombination of low-energy carriers, whilst retaining high-energy carriers to participate in catalytic reactions, clearly revealing the core logic that balances efficient carrier separation with strong redox activity [49].

Khan et al. used S-type carbon nitride (CN) homojunctions, which consisted of phosphorus-doped carbon nitride (P-CN) and boron-doped carbon nitride (B-CN), for the photocatalytic production of H_2_O_2_. As shown in Figure 9e, the S3 homojunction produced a H_2_O_2_ concentration of 2199.5 μmol L^−1^h^−1^ under LED_365nm_ irradiation. Structural and optical analysis confirmed the presence of B-P bond bridges between the P-CN and B-CN nanosheets. This B-P bridge causes an increase in the built-in electric fields. As shown in Figure 9f,g, the P-N bond energy in P-CN increases to higher binding energy, and the B-N/B-C bond energy in B-CN decreases to lower binding energy. This result shows that the B-P bridge acts as a “charge transport channel,” which helps to transport electrons from P-CN to B-CN. Consequently, a robust intrinsic electric field is established at the interface, effectively facilitating charge carrier separation and inhibiting recombination processes, thereby enhancing photocatalytic efficiency [51]. Likewise, Chen et al. designed a hydrogen-bond-induced carbon nitride (CN) homojunction (homo-CN). Figure 9h shows the mechanism of the photocatalytic production of H_2_O_2_ assisted by the homo-CN. Upon visible light irradiation of the CN homojunction, the interfacial electric field established at the junction boundary facilitates the electron transfer process from the conduction band of HD-CN towards HR-CN. As shown in Figure 9i, homo-CN exhibits the highest yield, at 508.4 μM. Additionally, the SA-TCPP nanocrystal (ST-NS/NC) homojunction catalyst, modified with tetra(4-carboxyphenyl)porphyrin (SA-TCPP) nanosheets, demonstrates a significantly enhanced H_2_O_2_ production activity due to its built-in electric field [52].

Furthermore, drawing on recent advancements in other catalytic fields, the use of magnetic single atoms to induce a “spin polarization effect” is emerging as a new strategy for enhancing carrier migration driven by an intrinsic electric field. For example, in recent research on photothermal catalytic CO_2_ methanation, researchers successfully induced a spin polarization effect by incorporating Mn single atoms onto the surface of hollow Ni_3_Si_2_O_5_(OH)_4_ nanotubes. This effect not only promotes the directional transfer of electrons and optimizes the adsorption of intermediates but also lowers the reaction energy barrier. This design approach—which utilizes “magnetic single atoms combined with spin polarization” to create an intrinsic electric field—provides highly insightful cross-disciplinary insights for overcoming the carrier separation bottleneck and driving efficient charge transport in photocatalytic H_2_O_2_ production systems [53].

#### 2.2.2. Defect Engineering

Defect engineering, concerning vacancy defects, metal doping, and distortion of electronic structure, can adjust the electronic structure of materials by introducing certain defects. This approach is employed to enhance the transport characteristics of photo-generated carriers and improve the efficiency of charge separation [54]. Kong et al. demonstrated that bulk defects act as deep traps, readily leading to ineffective charge recombination; surface defects, meanwhile, possess both shallow trapping and high adsorption properties, which can promote interfacial charge transfer and suppress recombination. By reducing the C_bd_/C_sd_ energy, bulk recombination centers can be eliminated, the average carrier free path extended, and surface charge output enhanced, thereby achieving efficient space charge separation and significantly improving photocatalytic performance [55]. At the same time, the concentration of surface defects is crucial for H_2_O_2_ production. A moderate concentration captures electrons and promotes two-electron reduction; an excessively high concentration not only leads to carrier recombination but also acts as an active site for the decomposition of the H_2_O_2_ that has already been generated [56].

**Figure 10:**
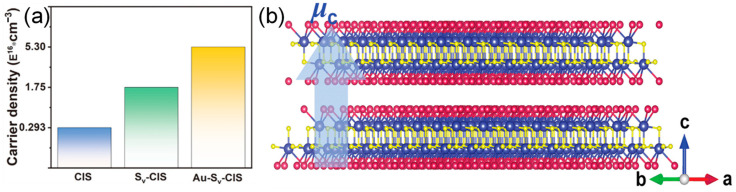
(**a**) Carrier concentration of the sample obtained through Hall effect testing [57]. Copyright 2025, Elsevier B.V. (**b**) Structural model of β-ZrNBr [58]. Copyright 2025, Cell Press.

Zhao et al. introduced charge separation by synthesizing CdIn_2_S_4_ (Sv-CIS) with high sulfur vacancies, followed by the deposition of gold nanoparticles on the surface (Au-Sv-CIS) to facilitate O_2_ adsorption. Sulfur vacancies possess the ability to effectively catch photons, thereby impeding the rapid recombination process of electron–hole pairs. As shown in Figure 10a, Hall effect analysis shows that this setup provides more charge carriers [57]. In addition to the creation of high-defect structures by vacancy engineering, materials such as β-ZrNBr can also create layered structures with low defect concentrations by creating low-defect crystal structures, as shown in Figure 10b. This approach will minimize charge trap sites and the possibility of carrier recombination. β-ZrNBr has high carrier mobility values (>100 cm^2^/V·s) and a bulk charge separation efficiency of over 70% [58]. Covalent organic frameworks (COFs) may face problems such as charge recombination during the photocatalytic reaction. The integration of fluorine atoms within the imine-linked pore structures of COFs enhances carrier separation efficiency and reduces recombination rates in HITMS-COF-20 and HITMS-COF-21 [59]. In the polyethyleneimine (PEI)-grafted indium sulfide (In_2_S_3_) with a high sulfur content, the presence of sulfur vacancies (S_V_) within the In_2_S_3_ structure gives rise to electron-rich domains. Through the optimization of electron transfer processes, these regions facilitate oxygen activation, thereby enabling the efficient conversion of adsorbed O_2_ into superoxide anions (·O_2_^−^) [60]. Drawing on interdisciplinary research strategies can also provide insights into the design of photocatalysts. For example, by incorporating the latest advancements in the field of electrocatalysis—specifically, the orbital hybridization between metals and specific crystal faces—it is possible to precisely induce and stabilize oxygen vacancies, offering a crucial interdisciplinary design approach for the fine-tuning of defect engineering in photocatalysis [61]. Furthermore, in biodiesel production, a novel heterogeneous bifunctional acid-base composite catalyst was developed by simply combining layered double hydroxide (LDH) active components with Cr-doped ZIFs-8 material (Cr-ZIFs-8/ZnCo-LDH). By improving the microstructure and enriching surface-active sites, this approach also significantly enhances catalytic efficiency [62]. Defect engineering can effectively modulate the electronic structure of catalysts, lower the energy barrier of key chemical bonds, and significantly enhance reaction kinetics [63]. This strategy of using defects to finely regulate surface reaction pathways in electrocatalytic systems offers significant inspiration for designing defect sites in photocatalytic H_2_O_2_ systems to promote oxygen activation and directed conversion.

### 2.3. Surface Redox Reactions

Surface redox reactions involve electron transfer reactions between photo-generated carriers and reactants at active surface sites. The surface redox reactions mainly involve the two-electron reduction of O_2_ (2e^−^ ORR), which is the major reaction. Meanwhile, the surface redox reactions suppress the four-electron reduction of O_2_ to H_2_O/O_2_^−^ (a side reaction) and the subsequent decomposition of H_2_O_2_ [64]. In fact, surface redox reactions not only determine the formation of H_2_O_2_ but are also the core causes of its instability. Mechanistically, the generated H_2_O_2_ is highly prone to secondary redox side reactions on the surface: on the one hand, H_2_O_2_ competes with O_2_ to capture photo-generated electrons on the surface, leading to its over-reduction to hydroxyl radicals (·OH) or water [65]. On the other hand, from a thermodynamic perspective, the standard electrode potential for the oxidation of H_2_O_2_ to O_2_ (0.68 V vs. NHE) is significantly lower than that for the oxidation of water (1.23 V vs. NHE). Therefore, in systems where the kinetics of water oxidation are slow, H_2_O_2_ is highly susceptible to preferential attack by strongly oxidizing photo-generated holes accumulated on the surface, leading to its re-decomposition into O_2_; this is another key factor contributing to its instability [66]. In particular, when surface catalytic sites lack spatial isolation, the disordered distribution of electrons and holes causes them to directly evolve into catalytic decomposition centers for H_2_O_2_ [23]. The photocatalytic reaction efficiency can be improved by optimizing the adsorption ability of O_2_, enhancing the selectivity of 2e^−^ reduction, reducing the charge recombination rate, and preventing the decomposition of H_2_O_2_. But the low charge transport rate and instability are still important issues that hinder the practical application of photocatalysts. In recent years, new strategies have been proposed to optimize the surface redox reaction efficiency to improve photocatalytic performance [67].

#### 2.3.1. Introduction of New Functional Groups

In photocatalytic H_2_O_2_ production studies, COFs have been identified as materials of choice for the introduction of new functional groups. As a new and rising family of new organic porous materials, the defining feature of COFs is the specific bonding of organic monomers by strong covalent bonds, yielding porous structures with high crystallinity and diversity [68]. Its tunable framework structure ensures a platform for accurate functional group implantation. The multi-porous properties and high specific surface area ensure efficient functional group activity with high stability to ensure the long-term durability of the photocatalyst. However, the main drawback of the traditional covalent organic framework (COF) photocatalyst for hydrogen peroxide production is the conflict between the proton and O_2_ demand and supply in the oxygen reduction reaction (ORR) and water oxidation reaction (WOR), which restricts the overall reaction efficiency [69]. As a major branch of the family of COFs, one-dimensional covalent organic frameworks (1D COFs) are defined by their structure, which are generated by the regular assembly of one-dimensional covalent bonds in a linear chain, as well as noncovalent forces such as van der Waals forces, π-π stacking, and hydrogen bonds that are oriented perpendicular to the two-dimensional and three-dimensional planes. Compared with 2D and 3D COFs, the dispersibility of 1D COFs is improved because of the reduced interchain and interlayer interactions, as well as the changed nonlinear edges and pore structures. This distinctive architecture not only enhances the accessibility of the active sites but also augments the application potential. One-dimensional COFs, such as EO-COF, ES-COF, and ESe-COF, are showing potential as low-cost H_2_O_2_ production materials. They possess well-ordered pore channels that promote oxygen diffusion and proton transfer, together with highly accessible active sites. These characteristics render them highly promising for diverse applications across multiple domains, such as catalysis, energy storage, adsorption, and sensing technologies [70].

**Figure 11:**
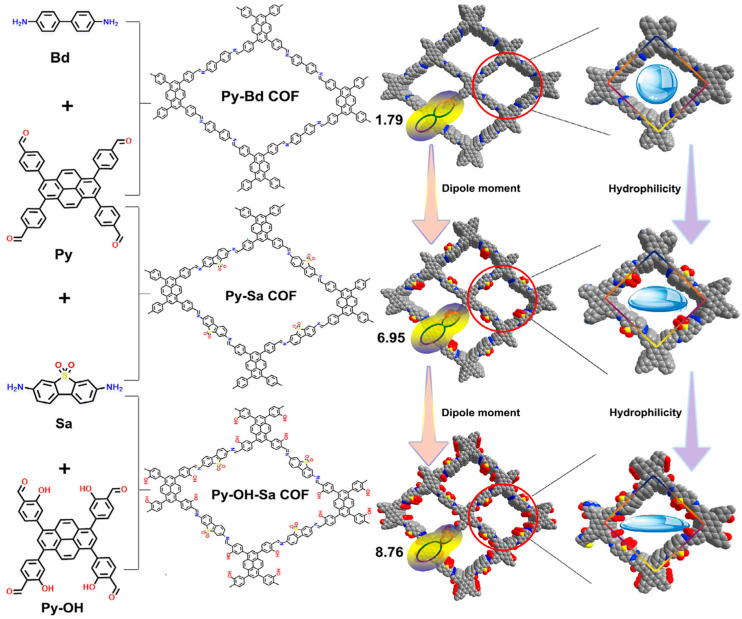
Schematic illustration of the synthesis and dual regulation of framework and microenvironment pore charge distribution in Py-Bd COF, Py-Sa COF, and Py-OH-Sa COF [72]. Copyright 2025, John Wiley & Sons, Ltd.

**Figure 12:**
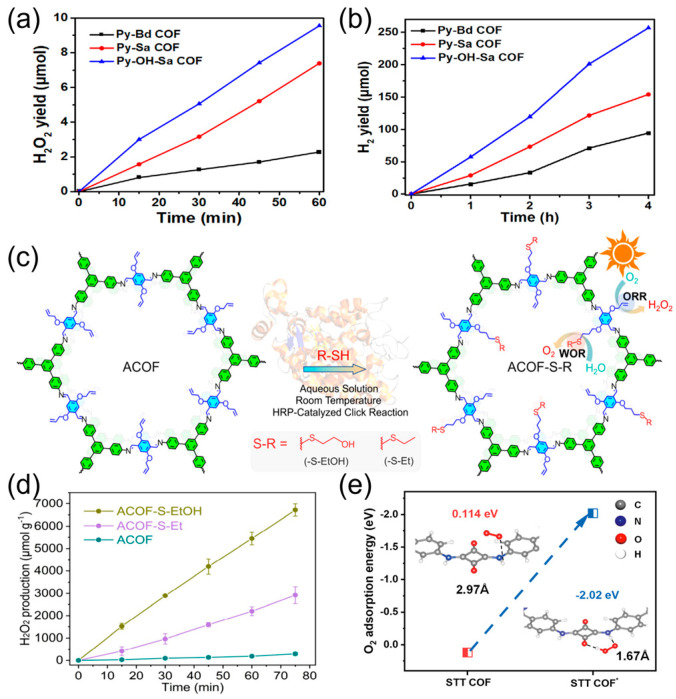
(**a**,**b**) Photocatalytic production of hydrogen peroxide and hydrogen over time [72]. Copyright 2025, John Wiley & Sons, Ltd. (**c**) Schematic of the enzymatic click reaction introducing -S-EtOH and -S-Et groups into COFs [73]. Copyright 2025, American Chemical Society. (**d**) Photocatalytic H_2_O_2_ production on different photocatalysts [73]. Copyright 2025, American Chemical Society. (**e**) Adsorption energies of O_2_ at optimal sites on STT COF and STT COF [74]. * indicates that oxygen is adsorbed on STT COF. Copyright 2025, Springer Nature Limited.

In redox reactions, there is a side reaction known as the four-electron oxygen reduction reaction (4e^−^ ORR), which produces water as its direct product. This side reaction greatly affects the efficiency of hydrogen peroxide (H_2_O_2_) production. The addition of new functional groups to the material will help in suppressing the side reaction, hence providing additional active sites for the ORR reaction [65]. Mu et al. prepared a covalent organic framework (COF) containing pyrazine units, it has a conjugated π system. They incorporated H_3_PO_4_ molecules into the COF, naming it N-heterocyclic TpDz. A hydrogen bonding network was formed between the H_3_PO_4_ molecules and the nitrogen atoms of the pyrazine units, which effectively formed a route in the COF pores to facilitate proton transfer. This strategy lowered the energy barrier of the ·ORR from ·ORR to H_2_O_2_ and improved the selectivity of the 2-electron ORR [71]. Likewise, as shown in Figure 11 below, Huang and colleagues synthesized covalent organic frameworks (COFs) through the simultaneous modulation of both the charge distribution across the framework and the microenvironment within the pores. The Py-OH-SaCOF was synthesized through a Schiff base condensation reaction involving Sa. The electron-withdrawing sulfonyl group and the electron-donating hydroxyl group in Py-OH-SaCOF facilitated the creation of more hydrophilic channels, which enabled efficient electron and proton transport to the photocatalytic sites. As a result, the hydrogen production rate reached 4.78 mmol g^−1^h^−1^ of H_2_O_2_ over time, as illustrated in Figure 12a,b [72].

The synthesis of N-heterocyclic TpDz was done by conventional post-synthetic modification (PSM), while the synthesis of Py-OH-SaCOF was done by reticular chemical design. Unlike the conventional methods of incorporating specific functional groups into covalent organic frameworks, Quan et al. introduced a new enzyme-catalyzed PSM method, which entails a click reaction catalyzed by horseradish peroxidase (HRP). Figure 12c shows the schematic representation of the enzyme-catalyzed reaction. Under aqueous media (room temperature and in the absence of toxic chemicals), 2-hydroxyethylthio (-S-EtOH) or ethylthio (-S-Et) groups are covalently bonded to the alkyloxy-functionalized pores of COF (ACOF), yielding ACOF-S-EtOH/ACOF-S-Et. This process has high grafting efficiency, is gentler, more effective, eco-friendly, and metal-free. The incorporation of the new 2-hydroxyethylthio (-S-EtOH) functional group further improves electron donation, the generation of hydrogenation intermediates, and the Gibbs free energy difference (ΔG), thus enhancing the rate of photocatalytic H_2_O_2_ production. As depicted in Figure 12d, the process exhibits a H_2_O_2_ generation rate of 5440 μmol g^−1^h^−1^ [73]. This innovative enzymatic method not only overcomes the challenges posed by the traditional methods in terms of environmental and efficiency issues but also sets a paradigm for the “molecular-level precision modification” of covalent organic frameworks (COFs).

Furthermore, the incorporation of novel functional groups could potentially enhance the density of active sites, optimize O_2_ adsorption capacity, and promote the direct occurrence of the two-electron oxygen reduction reaction (2e^−^ ORR) pathway. Chen et al. chose squaric acid (SQ) as the electron donor and 1,3,5-triazine-triphenylamine (TAPT) as the electron acceptor, and the resulting material was named STT COF. This material has an equimolar and ordered arrangement of cationic (C=NH^+^) and anionic (C-O^−^) functional groups, forming an amphoteric ion structure. As shown in Figure 12e, the hydrogenated STT COF* has active sites where the C=NH^+^ groups are located near the C-OH groups. These systems fix the Yeager-type adsorption structure of O_2_ by hydrogen bonding, which corresponds to the most favorable adsorption state for the 2e^−^ ORR process to form H_2_O_2_. During the reaction, the system with the lower ΔG in STT COF* favors the formation of H_2_O_2_ [74]. Likewise, a bis-sulfonic acid-functionalized covalent organic framework (2-SO_3_H-COF) was synthesized, which acts as an effective medium for proton transfer, thereby promoting solution-phase proton/O_2_ absorption and facilitating fast proton diffusion in the framework via a hopping mechanism. It is worth noting that the sulfonic acid group possesses dual-functional properties in proton regulation, where in proton-rich conditions, the -SO_3_H group acts as a ‘proton pump’ that attracts protons from the solution via strong polar interactions and facilitates fast proton diffusion to catalytically active sites via a hydrogen-bond network in the COF framework. In deprotonated conditions, the -SO_3_H group acts as a ‘proton reservoir’ that directly donates its protons to the ORR, thus ensuring that the reaction is not halted by a lack of protons [75]. The structural design and modification principles of functional groups based on COFs not only provide a clear approach for the precise regulation of the 2e^−^ ORR process but also provide evidence for the feasibility of optimizing the active sites and mass transfer properties by the strategic introduction of functional groups. This provides a crucial basis for the further improvement of highly efficient ORR catalysts.

Another catalyst which is based on covalent organic frameworks (COFs) is trifluoro pyridyl TFPy COF. The presence of fluorinated pyridine moieties has been found to greatly improve the hydrophilicity of the material, which in turn improves the dispersion and availability of catalytic sites in aqueous media [76]. Covalent organic frameworks containing hydroquinone units (Tz-QH-COF) significantly improve the stability of photocatalysts in H_2_O_2_ production [77]. Based on the success of C_3_N_4_ materials, functional heptazine moieties have been combined with other functional units into ordered molecular frameworks to improve hydrogen peroxide production abilities [78]. The hydrogen peroxide production ability may also be affected by the heteroatom locking in COFs. For example, N-heteroatom-locked COFs have a photocatalytic H_2_O_2_ production rate of 2.08 mmol g^−1^h^−1^ in pure water and air. This is due to the ‘locking’ effect, which increases coplanarity and conjugation, and the ‘heteroatom’ effect, which increases the adsorption capacity of O_2_ [79].

#### 2.3.2. Improving Redox Pathways

**Figure 13:**
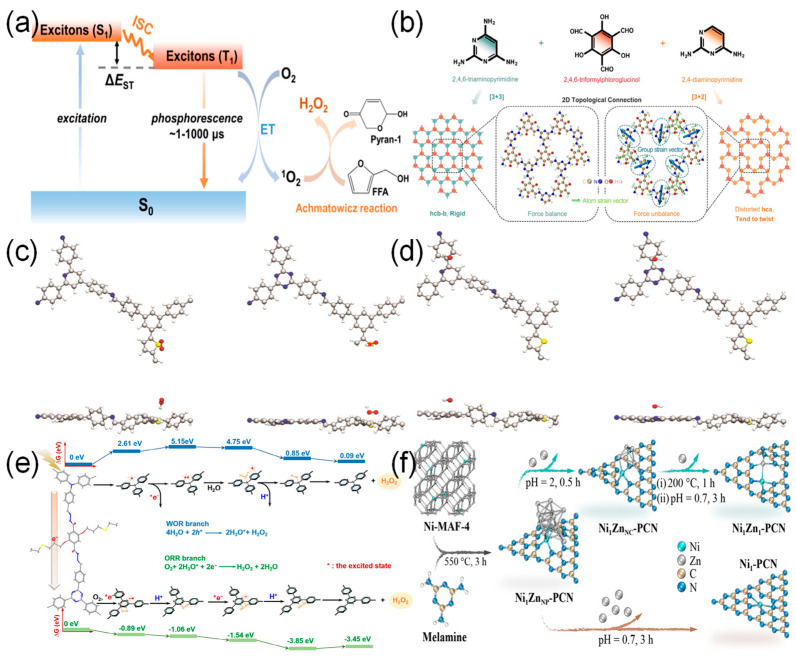
(**a**) Schematic pathway for photocatalytic H_2_O_2_ synthesis, illustration of H_2_O_2_ generation, and coupling of energy transfer processes with the Akhmatovich reaction [81]. Copyright 2025, John Wiley & Sons, Ltd. (**b**) Schematic configuration illustrating the topological tuning strategy for constructing twisted-geometry COFs; [3 + 3] denotes the 2,4,6-triformylphloroglucinol monomer bearing three -CHO groups linked to the 2,4,6-triaminopyrimidine linker; [3 + 2] denotes the 2,4,6-triformylphloroglucinol monomer with three -CHO groups linked to the 2,4-biaminopyrimidine monomer bearing two -NH_2_ groups [18]. Copyright 2025, John Wiley & Sons, Ltd. (**c**) The S atom in the thiophene ring of TD-COF and TT-COF serves as the active site for ORR. (**d**) The C atom in the benzene ring serves as the active site for WOR [82]. Copyright 2023, John Wiley & Sons, Ltd. (**e**) Gibbs free energy evolution and the proposed catalytic cycle diagram for ORR and WOR mechanisms in TBT-COF during the reaction [83]. Copyright 2025, John Wiley & Sons, Ltd. (**f**) Schematic of the two-step demetallation process for Ni_1_Zn_1_-PCN preparation [84]. Copyright 2025, American Chemical Society.

The traditional photocatalytic approach for H_2_O_2_ synthesis employs charge carriers generated from photon-induced processes as mediators for redox reactions. However, this technique is plagued by limitations, including inefficient charge separation and the inherent instability of H_2_O_2_, which renders it prone to rapid decomposition [80]. In Figure 13a, a carrier-free process was designed by combining energy transfer photocatalysis (EnTP) with the Akhmatovich reaction. In this process, O_2_ is activated to produce singlet oxygen (^1^O_2_) through energy transfer, thus bypassing the charge-dependent process in the traditional process, where O_2_ is reduced to superoxide anions by electrons. An A-CN catalyst was designed from the triazine framework by modifying the amide functional groups. This modification raised the exciton binding energy and narrowed the singlet-triplet energy gap, leading to improved efficiency and stability for H_2_O_2_ production [81]. As in Figure 13b, the pyrimidine-based COFs undergo a geometric transformation from a planar geometry (planar-PmTp-COF) to a twisted geometry (twist-PmTp-COF) by controlling the monomer linkage pattern. This geometric transformation is achieved by changing from a “3 + 3” planar linkage to a “3 + 2” asymmetric linkage. The geometric transformation is accompanied by a reaction mechanism involving triplet excitons and singlet oxygen (^1^O_2_), in which the produced triplet excitons directly excite ground-state oxygen (^3^O_2_) via triplet–triplet annihilation (TTA) [18]. However, these methods are very oxygen-dependent, and the efficiency of the reaction will decrease with lower concentrations of oxygen. Compared to catalysts that can follow both the ORR and the WOR pathways, these methods have lower environmental adaptability. These methods are difficult to apply in anoxic or hypoxic environments.

Yue et al. designed two new thiophene-based covalent organic frameworks (TD-COF and TT-COF) as catalysts for the synthesis of H_2_O_2_ via both indirect 2-electron ORR and direct 2-electron WOR. By adjusting the N-heterocyclic units in the COFs, Figure 13c,d show that the sulfur atoms in the thiophene units of TD-COF and TT-COF act as active sites for the ORR. However, the carbon atoms in the benzene units act as active sites for the WOR [82]. However, this reaction still has challenges concerning long-term stability, since the accumulation of H_2_O_2_ leads to the oxidation of the imine bonds of the catalyst, thereby deactivating it. It is worth noting that Han et al. carefully constructed the donors (triphenylamine, TN) and acceptors (triazine, TA) in COFs to create D-π-A structures. As shown in Figure 13e, the WOR on the TN moiety can interact with the ORR on the TA moiety. In addition, the TN moiety that takes part in the WOR can return to its original form during the reaction, thereby allowing for continuous photocatalytic activity [83]. In addition to the COF-based methods, the metal sites can be designed by dementalization strategies, in which one metal site assists in the electron capture for the ORR. Meanwhile, another metal site promotes the hole-steered WOR. As shown in Figure 13f, a dual-site Ni-Zn system was constructed in a PCN matrix by the N-coordination anchoring strategy, which assisted in the dual-pathway H_2_O_2_ catalysis with high stability [84]. Furthermore, co-doping Ce and Cr into MOF systems enables the construction of bifunctional systems with isolated acid and base properties; the strong synergy between the two metal sites significantly enhances the adsorption and activation efficiency of reactant molecules. In photocatalytic systems, this strategy of inducing microenvironmental synergy through bimetallic co-doping also holds great potential [85].

In redox pathways, the effective mass of a hole is greater than that of an electron, and its mobility is far lower than that of an electron; this means that the water oxidation half-reaction is often the rate-limiting step of the overall reaction. To accelerate the rate of the oxidation half-reaction, the difficult-to-proceed water oxidation reaction can be replaced with a thermodynamically more favorable organic oxidation reaction. However, most studies employ methanol or isopropanol, which are miscible with water. Whilst this achieves rapid hole consumption and enhances the production rate of hydrogen peroxide, it introduces new problems: The oxidation products of methanol or isopropanol are of low value; furthermore, the hydrogen peroxide generated mixes with these products, creating a significant challenge in terms of separation. A mixture of benzyl alcohol and water can be employed, which not only enables the efficient production of hydrogen peroxide but also allows for the selective oxidation of benzyl alcohol to produce the high-value chemical benzaldehyde. Following the reaction, the products automatically separate into layers by gravity, with benzyl alcohol and benzaldehyde distributed in the lower layer of the solution, whilst the aqueous hydrogen peroxide solution is distributed in the upper layer. For example, Ma et al. synthesized a novel antimony (Sb) and potassium (K) co-doped g-C_3_N_4_ (denoted as Cyano Sb Trz) via a tandem hydrothermal calcination strategy combined with a one-step molten salt method. By replacing the conventional sacrificial agent with benzyl alcohol, benzyl alcohol is oxidized via radical and non-radical pathways to form high-value-added benzaldehyde, whilst also establishing spatially separated redox active sites [15]. Using the Schiff base reaction, a superhydrophobic covalent organic framework photocatalyst with perfluoroalkyl functionalization was rationally designed and synthesized. This catalyst exhibits super hydrophobicity and spontaneously disperses in the oil phase, whilst the product H_2_O_2_ is extracted into the aqueous phase, forming a natural phase separation that facilitates separation [86].

**Figure 14:**
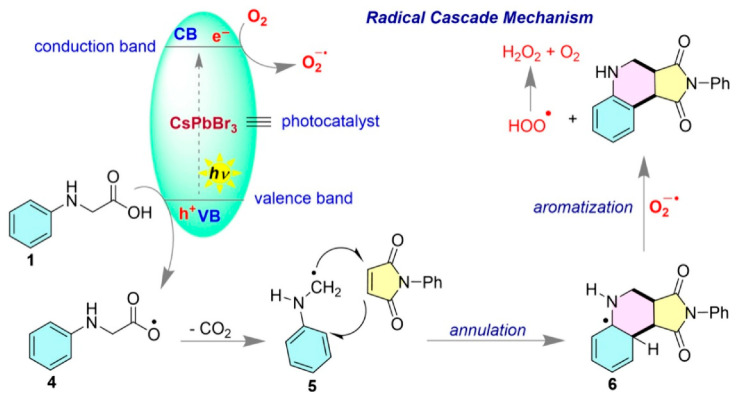
A plausible mechanism of the radical cascaded cyclization [88]. Copyright 2025 American Chemical Society.

Furthermore, in photocatalytic surface redox reactions, beyond simple water oxidation or oxygen reduction pathways, coupling the oxygen reduction reaction (ORR) with radical-mediated cyclization reactions in organic synthesis offers a highly promising new strategy for the generation of H_2_O_2_ and the co-production of high-value-added chemicals. For example, in photocatalytic synthesis systems involving nitrogen-containing heterocycles, the formation of hydroperoxyl radicals (HOO·) as intermediate species is involved. Take the radical-induced cascade cyclization reaction of N-phenylglycine with N-arylmaleimide catalyzed by CsPbBr_3_ nanocrystals as an example. As shown in Figure 14, in this reaction mechanism, N-phenylglycine is first oxidized by valence band holes generated by the photocatalyst. The resulting radical intermediate, after transformation and decarboxylation, reacts with N-arylmaleimide to form a condensation radical product; simultaneously, a superoxide radical (or HOO· formed via protonation) generated by the reduction of oxygen by conduction band electrons couples with this condensation radical in the final reaction step. This process simultaneously constructs the final product, 2-phenyl-3a,4,5,9b-tetrahydro-1H-pyrrolo [3,4-c] quinoline-1,3(2H)-dione, while releasing H_2_O_2_. This dual-channel coupling strategy not only effectively utilizes photo-generated carriers but also enables the synergistic production of high-value-added fine chemicals and green oxidants [87].

## 3. Enhance Mass Transfer

In the research of photocatalytic hydrogen peroxide production, three essential steps, including light capture efficiency, separation efficiency of photo-generated charge carriers, and surface oxidation reactivity, are important key points for improving reaction performance. But optimizing the conduction and adsorption properties of reactants, such as O_2_, on and in the surface of the catalytic material can effectively mitigate the restrictions of catalytic activity due to the lack of reactants. This measure can further improve the overall reaction kinetics of the catalytic system, providing another way to optimize performance in the efficient and stable production of H_2_O_2_.

### 3.1. Gas–Liquid–Solid Three-Phase Interface

In the conventional solid-phase catalysts, the interaction between the reactants takes place only through surface adsorption, making it difficult to coordinate gas-phase and liquid-phase reactants effectively. After the formation of hydrogen peroxide (H_2_O_2_), it tends to clog the active sites, thus slowing down the reaction. In gas-solid catalysts, there is a shortage of H_2_O supply, and gas-phase products are susceptible to decomposition. In solid–liquid catalysts, which are the most widely used systems at present, there is an abundant supply of proton sources. But the amount of oxygen (O_2_) in the liquid-solid interface is restricted by its solubility, making it difficult to further improve [89]. Conversely, the gas–liquid–solid three-phase interface not only overcomes the difficulty of slow O_2_ diffusion in the aqueous phase but also offers a plentiful proton source. This interface promotes the concentration of active sites, shows better efficiency for the separation of photogenerated charges, and has higher catalytic activity. Moreover, it enables the joint adsorption of O_2_ and H_2_O, thus activating the reaction [90]. A photocatalytic system characterized by a gas–liquid–solid three-phase interface, employing a CdS-deposited membrane, demonstrates the effective immobilization of the catalyst onto the filtration membrane [91], in another approach, the catalyst can be deposited onto hydrophobic carbon paper to establish a three-phase interface [92]. In addition, the sp^3^-hybridized nonpolar C-C single bonds can be used to combine the catalytic active units, so that the wettability of the photocatalyst can be directionally controlled, thus creating a three-phase interface [93]. Moreover, the covalent organic framework (COF) membrane with asymmetric wettability can be used. Based on the control of the wettability on both sides of the COF membrane, a three-phase interface can be formed, which favors the local enrichment of O_2_ and H_2_O, thus promoting the reaction efficiency [94].

**Figure 15:**
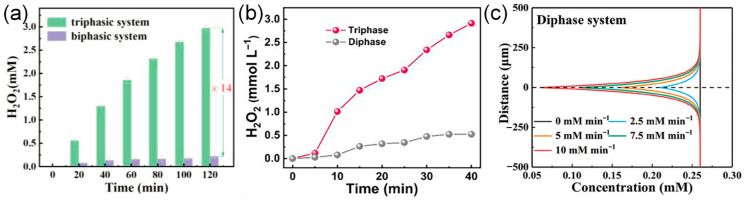
(**a**) Time course of H_2_O_2_ production under different reaction conditions in pure water under illumination [93]. Copyright 2024, John Wiley & Sons, Ltd. (**b**) Comparison of in situ H_2_O_2_ production in three-phase and two-phase systems [95]. Copyright 2022, Elsevier B.V. (**c**) Oxygen concentration in the diphase (the upper) and tri-phase system (the lower) calculated from diffusion model [96]. Copyright 2024, John Wiley & Sons, Ltd.

The conjugated organic polymer material (triazine-thiophene polymer, CTTP) proposed by Guo et al., under gas–liquid–solid three-phase conditions, the self-floating CTTP can rapidly capture gaseous oxygen, reduce it to superoxide radicals and transfer them to the reaction interface. As shown by the data in Figure 15a, this significantly increases the H_2_O_2_ production rate to 1.85 mM g^−1^h^−1^. The solid–liquid–gas three-phase reaction interface formed by CTTP with pure water and oxygen fundamentally resolves the issue of gas mass transfer in the oxygen reduction reaction, thereby enhancing the utilization of photo-generated electron pairs for oxygen reduction. The three-phase interface not only facilitates the fast production of H_2_O_2_ but also enhances the degradation efficiency of organic matter. As illustrated in Figure 15b, the Z-scheme heterojunction MIL-101(Fe)/g-CN_3_, developed by Ju, exhibited a H_2_O_2_ generation rate of 4370 μmol h^−1^ at the gas–liquid–solid tri-phase interface, resulting in a 17.5-fold enhancement in the organic matter degradation rate. The hydrophobic carbon cloth served as the substrate for loading the MIL-101(Fe)/g-C_3_N_4_ catalyst, thereby fabricating the composite catalyst designated as MIL-101(Fe)/g-C_3_N_4_/carbon cloth. The triphasic interface architecture of this catalytic system enhances oxygen concentration within the solution. The intrinsic hydrophobicity of the carbon cloth facilitates the diffusion of O_2_ from the ambient air to the photocatalytic interface, thereby establishing an optimal environment for the reaction. Additionally, the photogenerated electrons at the triphasic interface quickly reduce Fe^3+^ ions, produced during the reaction, to Fe^2+^ ions, thus facilitating continuous photo Fenton reactions [95]. In addition, through the preparation of COF membranes with asymmetric wettability, the asymmetry of the membrane creates a three-phase interface, which enriches O_2_ and H_2_O. The conversion of COF powders into nanostructured membranes optimizes light absorption properties and modifies the energy band configuration, thereby promoting water oxidation processes and amplifying photocatalytic efficiency. The study of asymmetry in this work opens up new ideas for the preparation of new COF materials [94].

In addition to providing qualitative evidence that the three-phase interface enhances H_2_O_2_ yield, multiphysics simulations can be used to model the O_2_ concentration at the interface in both two-phase and three-phase systems, thereby elucidating the mass transfer kinetics at the reaction interface. Yan et al. loaded polymeric carbonitride (C_V_-PCN), rich in pyrrole units and cyanide defects, onto a superhydrophobic melamine sponge modified with polytetrafluoroethylene (PTFE), thereby constructing a three-phase system based on a gas diffusion layer (GDL). Based on a diffusion model constructed using multiphysics simulation, the simulation results are shown in Figure 15c. The top layer shows the simulation results for the two-phase system; as O_2_ consumption in the catalytic layer increases, the O_2_ concentration at the interface decreases significantly. However, for the three-phase system (lower layer), as oxygen consumption increases, the reaction interface still maintains a high O_2_ concentration, approaching the standard solubility of O_2_ in water (0.26 mmol L^−1^). This phenomenon is attributed to the rapid diffusion coefficient of O_2_ in the GDL (2.0 × 10^−1^ cm^2^s^−1^), which effectively supplies O_2_ to the catalytic layer. Quantitative model calculations indicate that, thanks to this extremely rapid oxygen supply capacity, even at equivalent high consumption rates, the O_2_ concentration at the three-phase reaction interface can still be maintained at a level close to the saturation state of natural water (0.26 mmol L^−1^). This sustained and high-concentration local supply of O_2_ not only overcomes the mass transfer bottleneck but also enables O_2_ to act as an efficient natural electron scavenger, rapidly consuming photo-generated electrons to form ·O_2_^−^, thereby fundamentally suppressing the recombination of electron–hole pairs. Driven by this mechanism, the H_2_O_2_ yield of this system in pure water reached a benchmark level of 2063.21 μmol g^−1^h^−1^, which is 9.58 times that of powder catalysts and 5.68 times that of two-phase systems under equivalent conditions [96].

### 3.2. Reactor Design

The production of newly synthesized catalysts in reactors is a new and effective method of improving photocatalysis. Joint reactions can greatly improve the efficiency of photocatalysts in hydrogen peroxide production [97]. However, all the batch reactors can only work intermittently, requiring periodic separation and recovery of the catalysts every few hours to extract the H_2_O_2_ solution. This is because of low productivity and additional costs [86].

**Figure 16:**
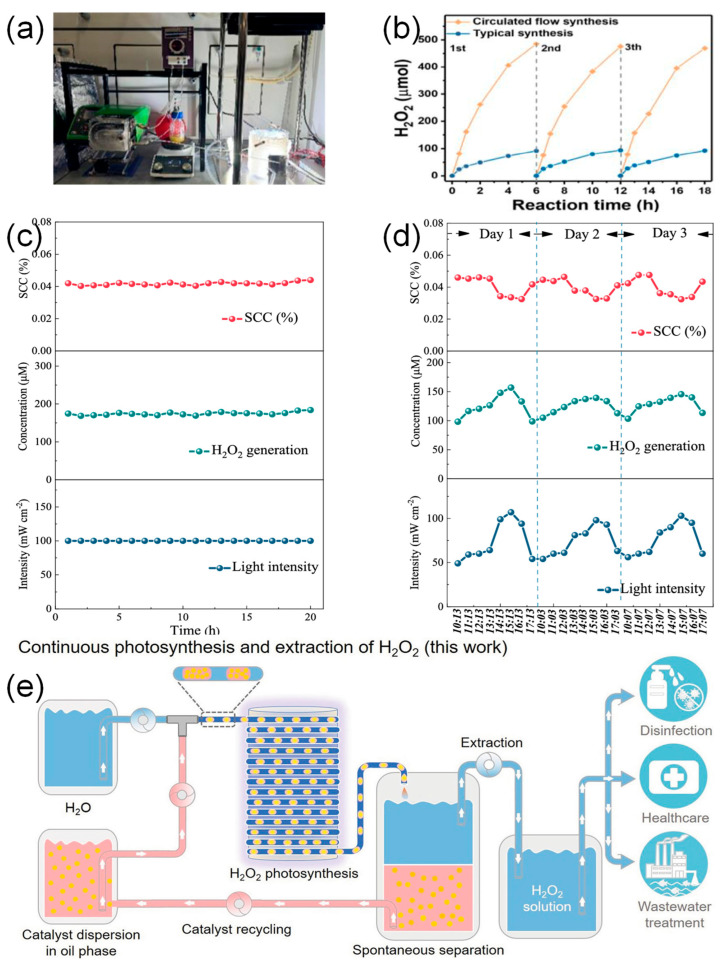
(**a**) Photograph of the PAQ-TABPB circulating flow synthesis system for H_2_O_2_ production. (**b**) H_2_O_2_ activity and circulation stability curves for the bath reaction system and circulating flow synthesis system [100]. Copyright 2025, John Wiley & Sons, Ltd. (**c**) Flow-based H_2_O_2_ production and solar-to-chemical (SCC) efficiency under simulated sunlight. (**d**) Flow-based H_2_O_2_ production and SCC efficiency under natural sunlight [101]. Copyright 2025, Springer Nature Limited. (**e**) A two-phase fluid system enabling continuous H_2_O_2_ photosynthesis, separation, and extraction. Yellow, blue, and pink represent the photocatalyst, aqueous phase, and oil phase, respectively [86]. Copyright 2024, Springer Nature Limited.

However, the use of circulating reactors and flow reactors in the production process can greatly improve the efficiency of photocatalysis and make it easier to apply in industrial production [98]. Cyclical reactions can improve mass transfer, light use, and the stability of reactions [99]. For example, a tri-monomer cross-linked polymer (PAQ-TABPB) was used to build a circulating reactor, referring to Figure 16a. In Figure 16b, the activity and stability of the circulating flow synthesis system are much higher than that of the bath reaction system. In addition, the alternate O_2_ bubbles and suspension droplets in the circulating reactor improve the mass transfer, which promotes the reaction efficiency [100]. The flow reactor is designed to enable the separation-free continuous production of H_2_O_2_. The reactor features a channel configuration filled with TpBpy, an imide-based covalent organic framework material, and employs a peristaltic pump to regulate the flow rate of ultrapure water. In the experiments, the flow rate was set at 2 mL min^−1^ to create a continuous and stable reaction system. The catalytic efficiency of hydrogen peroxide production was compared under natural and laboratory lighting. Refer to Figure 16c,d, generation rate of hydrogen peroxide in the laboratory-scale system was 11,429 mM h^−1^m^−2^, and the SCC efficiency was 0.040%. This efficiency was maintained in the absence of sacrificial agents. The process is simple and can be scaled up to satisfy the requirements of industrial continuous production [101]. Moreover, a biphasic water-oil reactor was also developed with the perfluoroalkyl-functionalized superhydrophobic covalent organic framework (PF-BTTA-COF) photocatalyst, as shown in Figure 16e. α,α,α-Trifluorotoluene (TFT) was chosen as the organic phase solvent. On the other hand, the distilled water was used in the aqueous phase reaction for the spontaneous extraction of H_2_O_2_, leading to a photocatalytic activity of 968 μmol h^−1^. It is pertinent to mention here that the symmetry mismatch in PF-BTTA-COF results in periodic uncondensed aldehyde site, which help in the stable dispersion of the photocatalyst in the oil phase of the two-phase fluid photoreactor [86]. Table 1 and Table 2 list the performance of photocatalytic hydrogen peroxide production reported in recent years.

## 4. Development Prospects

Although there have been many innovative approaches to the photocatalytic production of hydrogen peroxide, the field still faces numerous challenges. We have listed the issues facing photocatalysis in turn, summarized some examples that help address these issues, and outlined prospects for the future development of photocatalysis.

Certain semiconductor materials are prone to photodegradation under light exposure [7]; furthermore, as the H_2_O_2_ generated is itself a strong oxidizing agent, its accumulation within the reaction system readily oxidizes the active components or structural framework of the catalyst. For example, in covalent organic framework (COF) systems, accumulated H_2_O_2_ oxidizes and cleaves the imine bonds in the material, leading to structural collapse and deactivation of the catalyst, this results in poor stability of the photocatalyst [82]. Furthermore, during catalyst preparation or in two-phase flow reactors, organic solvents such as trifluoro toluene (TFT) must be used, which not only increases production costs but also poses a threat to the environment [86]. In traditional suspension-based reaction systems, most photocatalytic systems take the form of powder suspensions, which are difficult to recover and reuse; once they are released into the water cycle, they may pose environmental risks that are difficult to assess. The concentration of H_2_O_2_ generated via photocatalysis is generally low, far below the commercial standard of 1–3%, resulting in extremely high costs for the separation and purification of hydrogen peroxide from the product. Traditional batch reactors can only operate intermittently and require frequent shutdowns for catalyst separation, which leads to low productivity and makes it difficult to maintain system stability. However, when the system consists of a continuous-flow or multi-phase circulating reactor, it faces serious challenges regarding long-term operational stability: under prolonged gas–liquid–solid fluid erosion or thermal effects, catalyst coatings with insufficient interfacial adhesion are highly prone to mechanical delamination and extensive flaking, leading to a sharp decline in the reactor’s overall catalytic performance. Furthermore, in traditional suspension reactors, the depth of light penetration is extremely limited. In practical applications, catalysts in large reactors are unable to utilize light irradiation, and the low diffusion rate of oxygen in water leads to a decline in reaction efficiency. These are the engineering challenges that remain to be resolved [163].

To address the issue of photocatalyst stability, Ma et al. synthesized a carbon nitride material, Trz CN, possessing a massive built-in electric field (BEF) using a combined hydrothermal and calcination strategy. The massive BEF (4.8 times), induced by a large dipole moment, facilitated the efficient separation and directional migration of photo-generated carriers. Trz CN exhibits excellent cycling stability and remains stable in seawater environments [164]. This suggests that we can enhance BEF by developing new methods or introducing molecular units with large dipole moments, as well as by optimizing the hydrophilicity of the material to improve its stability in other environments. Through semi-solid polydimethylsiloxane (PDMS) interface engineering, the efficient immobilization of self-assembled porphyrin nanosheets (SA-TCPP) on quartz wool (QW) was achieved. The resulting PDMS/SA-TCPP/QW composite demonstrated stable photocatalytic performance over 10 reuse cycles, and in a modular flow reactor, the material continuously generated 3.5 mmol H_2_O_2_ over 50 h. This suggests that to enhance reactor stability, corrosion-resistant three-dimensional porous materials can be employed to encapsulate the catalyst and prevent its detachment; furthermore, modular reactors can be utilized to optimize fluid distribution and reduce maintenance costs. To address the difficulty in separating and purifying H_2_O_2_, a heterogeneous porous organic polymer (POP) photocatalyst has been developed, and an ‘oil-water’ two-phase reaction system has been established, utilizing lipophilicity to achieve spontaneous separation. Upon completion of the reaction, the mixed liquid enters a collector, where the two phases undergo spontaneous separation [165]. Selecting the ideal macroscopic carrier—quartz wool, PDMS is introduced as a physical encapsulation and bonding medium, forming a semi-solid coating on the surface of the quartz wool. Its viscosity firmly encapsulates the catalyst and physically anchors it within the quartz wool network. Furthermore, NH_2_-PDMS, containing amino end groups, reacts with the carboxyl groups on the surface of the SA-TCPP catalyst to form covalent amide bonds. This resolves the issue of organic catalysts readily leaching from the support, fundamentally eliminating the need for any powder separation steps such as centrifugation or filtration. This approach utilizes two-phase flow extraction and macroscopic immobilization to enable the spatial separation of the catalyst, substrate and products. To address the challenges of reactor scale-up for practical industrial applications, Li et al. achieved a robust and uniform dispersion of porous, nest-like mCN particles by loading them onto superhydrophobic polytetrafluoroethylene (PTFE) fibres, thereby providing a large surface area. This effectively minimized the likelihood of catalyst particles shading one another or becoming buried and avoided the issues of powder agglomeration and light attenuation in the deep water zone commonly encountered in suspensions [166]. The PDMS/SA-TCPP/QW composite prepared by Liu et al. innovatively concentrates the H_2_O_2_ produced to 100 mM via flow evaporation, demonstrating its potential for industrial-scale production. By coupling continuous flow evaporation with in situ separation of H_2_O_2_ to prevent oxidative degradation of the product, the system achieves highly efficient and stable large-scale application of the photocatalytic system [163]. Furthermore, expanding the scope of photocatalytic systems in the synthesis of complex, high-value-added organic compounds and pharmaceutical intermediates is also key to enhancing the overall economic efficiency of the system. Currently, visible-light catalysis has already achieved highly challenging transformations such as the directed radical nitration and ring-opening rearrangement of cyclobutanol [167]. Furthermore, recent research has successfully developed a hybrid photoelectrocathode based on non-precious metal molecular catalysts, achieving the efficient conversion of CO_2_ into high-value hydrocarbons under solar drive [168]. In future reactor and catalyst design, exploring the compatibility and coupling of efficient photosynthesis of complex pharmaceutical intermediates with systems for the conversion of challenging carbon resources and the green production of H_2_O_2_ will represent a significant leap toward industrial-scale applications in this field. Furthermore, developing “waste-to-waste” material synthesis strategies is also an important future research direction. For example, Wang et al. proposed a sustainable engineering approach that innovatively utilized red mud as a feedstock to prepare FeOOH/BiVO_4_ composite photoanodes, thereby transforming solid waste into highly efficient catalysts [169]. In the future, in-depth exploration of the resource utilization potential of various industrial solid wastes in the photocatalytic synthesis of H_2_O_2_ will greatly advance the low-cost implementation and commercialization of this technology.

In the areas of material science and nanotechnology, the accurate observation of the crystal structure at the atomic level is very important, and it depends largely on the advancement of electron microscopy technology [170]. The direct evidence for comprehending the influence of these defects on the electrical and optical characteristics of two-dimensional materials lies in the identification of single-atom vacancy defects. These defects arise from the absence of atoms and the dislocation defects triggered by atomic misalignment. This serves as the foundation for uncovering novel photocatalysts [171]. Furthermore, the expansion of in situ dynamic characterization scenarios and the simulation of actual reaction environments are important methods for understanding catalytic mechanisms and improving material properties [172]. The ability of dynamic characterization has broken through the constraints of traditional static characterization, providing direct experimental evidence for the correct interpretation of the “structure–property” relationship and the discovery of actual active sites. This advancement enables rational catalyst design informed by fundamental mechanisms [173]. In particular, the combined application of femtosecond/picosecond transient absorption spectroscopy (TAS), time-resolved photoluminescence (TRPL) and in situ electron spin resonance (ESR) techniques should be more widely employed in the future to track the entire charge transfer pathway from photon absorption to H_2_O_2_ generation. This will provide the most direct physicochemical evidence for overcoming the kinetic bottlenecks of the reaction and elucidating the complex multi-electron redox rate-determining steps at multiphase interfaces. The traditional characterization techniques, such as X-ray diffraction (XRD), high-resolution transmission electron microscopy (HRTEM), and Raman spectroscopy, have the ability to identify microscopic details. However, these techniques require manual analysis, which may lead to low efficiency and high subjectivity. The combination of content that enables fast machine identification of defects and crystal orientations can greatly improve the efficiency of analysis. This allows the construction of quantitative ‘defect activity’ models by big data mining, which can guide the rational design of the catalyst [174]. For example, Wang proposed a machine learning framework based on in situ infrared spectroscopy. This study moved away from traditional trial-and-error methods by directly extracting the intensity of infrared peaks corresponding to intermediates formed by the adsorption of NO molecules on the catalyst surface as data features. Using gradient-boosted regression (GBR) and random forest regression (RFR) algorithms, it successfully predicted the yield of nitrate products following illumination and the final NO removal efficiency. This machine learning approach reduced experimental time by a factor of 3.5 and enabled the quantitative determination of catalytic performance [175]. Vijay C. Karad employed a machine learning (ML)-guided strategy to optimize the doping concentration of germanium (Ge) to enhance the benchmark performance of CZTSSe. The machine learning model indicated that a Ge doping concentration of less than 5% is optimal for achieving higher device performance and reducing open-circuit voltage (VOC) loss, and that controlling the carrier density by regulating defects plays a key role in the fabrication of high-quality devices [176].

On the other hand, computer calculation based on limited experimental results can also be used to rapidly determine the best parameters in the synthesis process. Most previous research studies have used single-factor experiments. By using machine learning to construct complex nonlinear relationships between the descriptors and the properties, it is also possible to make precise predictions of the properties of the catalyst and rapidly optimize high-dimensional parameters. For example, supervised learning models such as support vector machines (SVMs), artificial neural networks (ANNs) and tree-based ensemble algorithms (such as random forests) can be used to accurately construct non-linear predictive models relating operating conditions to pollutant degradation efficiency; their predictive accuracy (R^2^) often exceeds 0.95, providing crucial theoretical support for the use of photocatalysis in the production of H_2_O_2_ [177]. At the same time, the goal is not merely to predict catalyst synthesis, but also to understand the underlying catalytic mechanisms and thereby identify applicable patterns. Guo et al. introduced a dual-engine AI framework that integrates closed-loop active learning with interpretable machine learning. Within this framework, the discovery engine autonomously explores the vast catalyst space by combining density functional theory with experimental feedback, extracting human-understandable physical descriptors and “transparent box” design principles from massive datasets. Applying this strategy—which integrates theoretical screening with in-depth mechanistic analysis—to the field of photocatalytic H_2_O_2_ will significantly accelerate the discovery and translation of novel, highly efficient catalysts [178].

## Figures and Tables

**Table 1:** Comparison of different catalyst performances (no sacrificial agent).

Material	Sacrificial Agent	Atmosphere	Irradiation Conditions	Rate (mmol g^−1^h^−1^)	Activity Retention	Ref
Ni_1_Zn_1_-PCN	H_2_O	O_2_	AM 1.5G	1.205	Activity well-retained after 5 cycles	[84]
MQ-BT	H_2_O	O_2_	λ > 420	7.095	No obvious yield decreases after 5 cycles	[102]
OH-COF-E	H_2_O	Air	λ > 420	1.987	No obvious yield decreases after 5 cycles	[103]
PD-TP	H_2_O	O_2_	λ > 420	12.100	Excellent stability after 5 cycles	[104]
	H_2_O	Air	λ > 420	10.160	Excellent stability after 5 cycles	
BTT-MD-COF	H_2_O	O_2_	λ > 420	5.690	No obvious yield decreases after 4 cycles	[105]
COF-N32	H_2_O	Air	λ > 420	0.605	No obvious activity loss after 5 cycles	[106]
	H_2_O	Air	Sunlight	0.667	No obvious activity loss after 5 cycles	
COF-2CN	H_2_O	O_2_	λ > 420	1.601	No obvious activity loss after 5 cycles	[107]
CNIO-GaSA	H_2_O	O_2_	λ > 420	0.332	Activity well-retained after 5 cycles	[108]
U6N@ZIS	H_2_O	Air	λ > 400	3.200	No obvious yield decreases after 5 cycles	[109]
	H_2_O	O_2_	λ > 400	3.800	No obvious yield decreases after 5 cycles	
USTB-46	H_2_O	O_2_	λ > 420	8.274	Activity remained stable after 4 cycles	[110]
SO_3_H-EB-COF	H_2_O	O_2_	λ > 420	1.730	No obvious activity loss after 5 cycles	[111]
	H_2_O	Air	λ > 420	0.940	No obvious activity loss after 5 cycles	
CoO_X_-BCN-FeOOH	H_2_O	O_2_	λ > 420	0.340	Yield remained stable after multiple cycles	[112]
	H_2_O	N_2_	λ > 420	0.020	Yield remained stable after multiple cycles	
TAPT–FTPB COFs	H_2_O	O_2_	Sunlight	3.780	No significant activity decrease after multiple cycles	[113]
PI-BD-TPB	H_2_O	O_2_	AM 1.5G	2.833	Yield remained stable after long-term cycling	[114]
FS-COFs	H_2_O	Air	λ > 420	3.904	Yield remained stable after multiple cycles	[115]
PB-COF	H_2_O	O_2_	AM 1.5G	2.044	No obvious yield decreases after 5 cycles	[116]
COF@H_3_PO_4_	H_2_O	O_2_	λ > 420	0.706	No obvious yield decreases after 5 cycles	[117]
BTDB-CN_0.2_	H_2_O	Air	λ > 420	1.920	No obvious decrease after 4 cycles	[118]
Kf-AQ	H_2_O (pH = 13)	O_2_	λ > 400	4.784	No obvious yield decreases after 5 cycles	[119]
DT-COF-1	H_2_O	O_2_	λ > 420	4.300	Negligible decay after 5 cycles	[120]
CdS/NiPc-10%	H_2_O	Air	λ > 420	34.400	Activity remained stable after multiple cycles	[121]
TZ-COF	H_2_O	O_2_	λ > 420	6.800	Activity well-maintained after 5 cycles	[122]
COF-343	H_2_O	Air	λ > 420	2.980	No obvious activity decrease after 5 cycles	[123]
Thz-COF	H_2_O	O_2_	λ > 420	82.300	Maintained excellent stability after 5 cycles	[124]
DFH-COF	H_2_O	O_2_	λ > 400	6.075	Yield well-maintained after 5 cycles	[125]
	H_2_O	Air	λ > 400	3.385	Yield well-maintained after 5 cycles	
TfpBQ	H_2_O	O_2_	electro-catalysis 120 mA cm^−2^	19,300	No obvious decay in yield and efficiency during long-term continuous electrolysis	
Ni-PCN-Co_2_P	H_2_O	O_2_	λ > 420	0.034	No significant activity decrease after multiple cycles	[126]
TAS_4_	H_2_O	Air	AM 1.5G	4.302	No obvious yield decreases after 5 cycles	[127]
	H_2_O	Air	λ > 420	3.500	No obvious yield decreases after 5 cycles	
TP-DBS- COF/ZSM-5-15%	H_2_O	O_2_	λ > 420	1.420	Yield remained stable after 5 cycles	[128]
PTA/g-C_3_N_4_	H_2_O	Air	λ > 420	2.491	No significant activity decay after multiple cycles	[129]
ATP-COFs	H_2_O	O_2_	λ > 420	14.000	Production rate basically unchanged after 5 cycles	[130]
CNB/CF	H_2_O	O_2_	λ > 420	0.936 (mmol m^−2^h^−1^)	Excellent stability in long-term continuous/cycling tests	[131]
Au_18_NAC_14_-TpBpy	H_2_O	O_2_	AM 1.5G	13.800	Performance is highly stable after multiple cycles	[132]
TAPT-DHBD COFs	H_2_O	O_2_	AM 1.5G	2.701	No obvious activity decreases after multiple cycles	[133]
TpDt	H_2_O	O_2_	λ > 420	6.499	Excellent stability after 5 cycles	[134]
T-ZrN	H_2_O	Air	electro-catalysis	55,600	Excellent stability after 5 cycles	
TFBP-DHBD COF	H_2_O	Air	AM 1.5G	1.444	No obvious yield decreases after 5 cycles	[135]
TPM-DADK	H_2_O	Air	AM 1.5G	17.220	Activity well-retained after multiple cycles	[136]
Ni-CDs/COFs	H_2_O	Air	VIS	11.603	Activity well-retained after 5 cycles	[137]
CityU-45	H_2_O	O_2_	λ > 420	4.854	Activity well-retained after multiple cycles	[138]
TPM-DADK (before exfoliation)	H_2_O	O_2_	λ > 420	4.720	Activity well-retained after 5 cycles	[136]
TPM-DADK (after exfoliation)	H_2_O	O_2_	λ > 420	10.820	Activity well-retained after 5 cycles	[136]
g-C_3_N_4_	H_2_O	O_2_	λ > 420, AM 1.5G 100 mW cm^2^	0.206	Activity remained stable with no significant decay after 5 cycles	[139]
g-C_3_N_4_-B10	H_2_O	Air	λ > 420, AM 1.5G 100 mW cm^2^	2.952	Activity remained stable with no significant decay after 5 cycles	[139]
CZ-AQ (inter-ring bonding)	H_2_O	Air	λ > 400	4.401	Excellent stability with no obvious activity decrease after 5 cycles	[140]
CZ-1-AQ (no alkynyl linker)	H_2_O	Air	λ > 400	1.657	Excellent stability with no obvious activity decrease after 5 cycles	[140]
Tp-TTz COF	H_2_O	O_2_	Full spectrum (300 W Xe lamp), 25 °C	1.500	Performance and structure remained highly stable after multiple cycles	[141]
TpPm (pyrimidine-based)	H_2_O	O_2_	λ > 420, 200 mW cm^−2^	17.014	Activity well-retained with no obvious deactivation after 5 cycles	[142]
TpMp (benzene-based)	H_2_O	Air	λ > 420, 200 mW cm^−2^	1.556	Activity well-retained with no obvious deactivation after 5 cycles	[142]
PDA/SA-TCPP	H_2_O	O_2_	Visible + NIR light (λ > 420 nm, 200 mW cm^−2^)	0.421	Excellent stability and stable yield after 5 cycles under NIR light	[143]
PDA/SA-TCPP	H_2_O	O_2_	saturated NIR light (λ > 800 nm, 80 mW cm^−2^)	0.124	Excellent stability and stable yield after 5 cycles under NIR light	[143]

**Table 2:** Comparison of different catalyst performances (with a sacrificial agent).

Material	Sacrificial Agent	Atmosphere	Irradiation Conditions	Rate (mmol g^−1^h^−1^)	Activity Retention	Ref
MQ-BT	10% BA	O_2_	λ > 420	16.892	No obvious yield decreases after 5 cycles	[102]
OH-COF-E	10% TEOA	Air	λ > 420	7.308	Performance well-maintained after 5 cycles	[103]
CTF-BTT	10% BA	O_2_	λ > 420	74.956	Activity well-retained after 5 cycles	[144]
TZ-COF	50% BA	Air	λ > 420	4.951	Performance well-maintained after 5 cycles	[145]
TiO_2_/MoS_x_-Au	10% ethanol	O_2_	300 W Xe lamp	30.440	No obvious activity decay after 4 cycles	[146]
COF-300-D-F	10% BA	Air	λ > 420	19.090	Performance well-maintained after 5 cycles	[147]
CdS/NiPc-10%	10% Isopropanol	O_2_	λ > 420	34.400	Activity well-maintained after 5 cycles	[121]
CdS/NiPc-10%	10% IPA	O_2_	λ > 420	41.300	Activity remained stable after multiple cycles	[121]
TP-DBS-COF/ZSM-5-15%	10% BA	O_2_	λ > 420	2.960	Yield remained stable after 5 cycles	[128]
PTA/g-C_3_N_4_	10% BA	Air	λ > 420	2.987	No significant activity decay after multiple cycles	[129]
O_V_-Bi_2_O_2_S/Co_9_S_8_	GLY	O_2_	λ > 420	52.820	No obvious performance decrease after multiple cycles	[148]
BTD-BTT-COF	Acetonitrile system (containing 5 mM benzylamine)	O_2_	λ > 420	25.670	No obvious activity loss after 5 cycles	[149]
H-NiMn_2_O_4-β_/Au_0.5_NCs	10% BA	O_2_	Xe	1.000	No significant yield decay after continuous cycling	[150]
COF-N_0_	Furfur alcohol (10 mM L^−1^)	O_2_	λ > 420	4.549	No obvious yield decreases after 5 cycles	[151]
Fe SAS-TpPP-COF	10% BA	O_2_	AM 1.5G	4.130	Activity well-retained after 5 cycles	[152]
In_2_S_3_-V	10% TEA	O_2_	λ > 420	4.770	No obvious yield decreases after 5 cycles	[153]
BTT-AQ	10% BA	O_2_	λ > 400	27.426	Excellent stability with well-maintained activity after 5 cycles	[154]
TANB-Py-COF	1.0 eq. Benzylamine 2 mol% COF	Air	Λ = 410	18.320	Activity well-retained after 5 cycles	[155]
BiOBr/g-C_3_N_4_	Benzaldehyde and water	O_2_	AM 1.5G	1.770	Outstanding stability after 5 interfacial cycles	[156]
Bi_2_WO_6_	50% BA	O_2_	AM 1.5G	121.530	Outstanding stability after 5 interfacial cycles	[157]
CdS/Bi_2_WO_6_	50% BA	O_2_	AM 1.5G	216.760	Outstanding stability after 5 interfacial cycles	[158]
IPr_0.5_@Ag_1_/TiO_2_	FA	O_2_	AM 1.5G	15.060	No significant deactivation after 5 cycles	[159]
PCHA	methanol	Air	λ > 400 100 mW cm^2^	0.163	Activity well-retained after 5 cycles	[160]
PCHA-SO_3_H	methanol	Air	λ > 400 100 mW cm^2^	2.277	Yield remained stable after 5 cycles	[160]
NC@TiO_2_	10% methanol	O_2_	Full spectrum (Xe lamp, 0.4 W cm^−2^, pH = 3, 6 °C)	350.500	Activity well-retained after long-term/cycling tests	[161]
RD@TiO_2_	10% methanol	O_2_	Full spectrum (Xe lamp, 0.4 W cm^−2^, pH = 3, 6 °C)		Activity well-retained after long-term/cycling tests	[161]
Pt_1_Au_5_/TiO_2_	methanol	2.9 MPa 5% H_2_/N_2_ 1.1 MPa 25% O_2_/N_2_	No light	45.100	No obvious yield decrease after multiple reuses	[162]
	methanol	2.9 MPa 5% H_2_/N_2_ 1.1 MPa 25% O_2_/N_2_	No light	128.600	No obvious yield decrease _after_ multiple reuses	[162]
Tp-TTz COF	10% BA	O_2_	Full spectrum (300 W Xe lamp, 100 mW cm^−2^), water/BA = 9:1 (*v*/*v*)	100.900	Performance and structure remained highly stable after multiple cycles	[141]

## Data Availability

Data is contained within the article.
